# A protocol of a randomized control trial to test the feasibility and efficacy of the EMPOWER social-emotional learning curriculum for youth aged 11–14 years in after-school settings

**DOI:** 10.1371/journal.pone.0319398

**Published:** 2025-03-17

**Authors:** Alice-Simone Balter, Madison Moloney, Clement Ma, Alina Lee, Sandra Pierre, Sheldon Parkes, Doga Pulat, Nicole Racine, Brendan F. Andrade

**Affiliations:** 1 Margaret and Wallace McCain Centre for Child, Youth and Family Mental Health, Centre for Addiction and Mental Health, Toronto, Ontario, Canada; 2 Biostatistics Core Services, Centre for Addiction and Mental Health, Toronto, Ontario, Canada; 3 Dalla Lana School of Public Health, University of Toronto, Toronto, Ontario, Canada; 4 Toronto Foundation for Student Success, Toronto, Ontario, Canada; 5 Department of Psychology, University of Ottawa, Ottawa, Ontario, Canada; 6 Department of Psychiatry, University of Toronto, Toronto, Ontario, Canada; PLOS: Public Library of Science, UNITED KINGDOM OF GREAT BRITAIN AND NORTHERN IRELAND

## Abstract

**Introduction:**

Promoting youth mental health and well-being is a global concern. Administering social-emotional learning programs in contexts that are familiar to youth have the potential to increase mental well-being by helping youth develop fundamental coping skills that may contribute to their resilience. Implementing social-emotional learning programs in after-school settings is a unique opportunity to improve mental well-being skills in communities that face inequities.

**Methods:**

The study is a partnership between investigators at an academic mental health hospital and an after-school program embedded within economically and socially vulnerable neighborhoods in a large metropolitan city in Ontario, Canada. This 20-week covariate-constrained randomized controlled trial will test the feasibility and preliminary efficacy of the EMPOWER social-emotional learning curriculum for youth aged 11-14 years in an after-school program. Twenty sites will be randomized to an intervention group or no-intervention control. Program staff in the intervention arm will receive training on the manualized curriculum and weekly coaching sessions to build capacity and support implementation over the 16-week program. Program staff and youth across both intervention and no-intervention control groups will be asked to participate in baseline and post-intervention data collection where they may complete questionnaires about youth’s social-emotional learning skills, global quality of functioning, and resilience skills. The no-intervention control group will carry on with their regular programming while the intervention group implements the 16-week social-emotional learning curriculum, after the collection of baseline data. Program staff in the intervention group will be asked to complete weekly fidelity measures and monthly feasibility, acceptability, and appropriateness of implementation scales. Parents/caregivers of youth in the intervention group will be asked to participate in a brief interview to report their observations of their children’s social-emotional learning skills.

**Dissemination:**

Results from this pilot study will be disseminated in a peer-reviewed journal and at community and academic conferences.

## Introduction

Youth mental health has been on the decline for more than a decade [1,2] and was labelled a public health priority in Canada in 2018 [3]. The early onset of mental disorders in childhood and adolescence coupled with a lack of access and barriers to mental health services is alarming [3,4]. A meta-analysis of 29 studies from across the globe illustrates a remarkable increase in the prevalence of clinical anxiety (25.2%) and depression (20.5%) during the pandemic [5]. These percentages do not capture children and youth who are below diagnostic thresholds and may also be struggling with their mental well-being, including those from communities that are socially and economically vulnerable.

Racialized and marginalized populations experienced disproportionate mental health challenges during the COVID-19 pandemic [5,6]. Although “race-based data related to all health outcomes are not collected in Canada” [7] p116) there is evidence from the US that Black youth experience disparities in some mental health disorders (e.g., major mood disorders) [7]. A social determinants of health framework [8] explains how “social factors may be precipitating, provoking, or perpetuating” [9] p864) negative mental well-being. A lack of, and barriers to, accessing mental health services exists for youth in general [3] and for Black and marginalized youth specifically [10], leaving many youth unsupported. Community-based mental health prevention and promotion programs have been shown to increase coping and resilience skills and provide more equitable access to mental well-being supports because they are embedded within settings that children and youth already access [11].

## Social emotional learning programming

Community-based social-emotional learning (SEL) programs aim to increase skills that build resilience for all children and adolescents. These SEL skills are critical building blocks to strengthen well-being in childhood, adolescence, and beyond [12]. The Collaborative of Academic, Social and Emotional Learning (CASEL) outlines an evidence-based SEL framework that consists of inter- and intra-personal skills that are couched within classroom, family, school, and community environments. There are five core SEL competencies that include self-awareness, self-management, social awareness, relationship skills, and responsible decision-making. *Self- awareness* focuses on the recognition and comprehension of thoughts, feelings, values, biases, personal, linguistic and cultural strengths, self-efficacy, and the interactions between these attributes [13]. *Self-management* consists of regulating affect, thoughts, and actions by managing stress and applying emotion management strategies to develop internal sources of discipline, motivation, and initiative [13]. *Social awareness* relates to discerning the viewpoints of others from diverse backgrounds and contexts, displaying empathy, and showing gratitude [13]. *Relationship skills* are comprised of effective communication, collaborative problem-solving, and asking for and providing help [13]. Finally, *responsible decision-making* involves learning how to think critically and develop problem-solving strategies, understand and evaluate one’s responsibilities and the consequences of action or inaction, and showcasing open-mindedness [13], and is constructed as a domain that informs the pedagogy of SEL [14].

Building SEL skills throughout childhood and adolescence is opportune, as these early years are a sensitive developmental period where the onset of mental disorders emerge and simultaneously when positive mental well-being skills can be nurtured to help mitigate the development of mental health difficulties [4]. It is during this time of neurological and physiological change that children and adolescents can build on previous, or develop new, SEL skills that act as protective factors [15,16]. SEL programs are associated with beneficial outcomes for youth, including improvements in (a) personal skills, such as self-regulation, responsible decision making self-perception, positive attitudes towards others; (b) social skills, such as the prosocial behaviors of cooperation and helping others and social awareness, including empathy, and (c) academic performance, as well as (d) reductions in substance use, (e) conduct problems, and (f) emotional distress [11,17,18]. According to a systematic review of studies of 32 international and universal SEL programs, adolescents aged 11-19 improved significantly across all five SEL domains, with the greatest effects in social awareness (e.g., taking the perspective of others and emphasizing as well as withstanding pressure from peers and solving conflicts), followed by self-awareness skills (e.g., self-efficacy, awareness of one’s emotions and thoughts), and the smallest effects in relationship skills [19]. More recent meta-analytic findings on more than 500,000 students from grades K-12 participating in SEL programs demonstrate enhanced prosocial behaviors, self-efficacy, perseverance, self-esteem, moral reasoning, performance in school and reductions in emotional distress bullying, noncompliance difficulties, and aggression [20]. SEL skills have been shown to be retained for up to 3.75 years and predict well-being improvements into adulthood, irrespective of geographic, socioeconomic, or racial [18,21]. Despite these promising findings, it is important to recognize that the benefits of SEL programs can be biased against lower income communities as schools and after-school programs often experience resource shortages [22], which may limit access to SEL programs. Embedding SEL programming into after-school settings in marginalized communities is especially important when considering inequities in the social determinants of health that influence well-being on a community level [23,24], as they provide a unique opportunity to improve access to increased mental well-being.

## The EMPOWER project

The EMPOWER project is a community-academic partnership between two after-school programs that operate in marginalized neighborhoods with a large majority of Black and racialized families, and an academic mental health hospital in a large metropolitan city in Canada. EMPOWER was established during the pandemic because youth in these broad communities were experiencing challenges with their mental well-being. The main goal of our partnership is to improve youth mental well-being by developing and embedding an SEL curriculum into these after-school programs. We have detailed the process of our partnership [25], are currently preparing a manuscript that details our curriculum development process [26], and published research that informed curriculum co-development [27,28].

The EMPOWER SEL curriculum was tailored to after-school settings and addresses some of the barriers of SEL implementation illustrated in the literature. Although SEL programs have been shown to improve key youth developmental outcomes [17,18,29], several barriers can make the implementation of SEL programming unsuccessful or unsustainable. First, implementing an SEL program in an after-school setting requires a significant level of organizational buy-in and financial aid to support the effective delivery of the curriculum [30] and fit [31]. Furthermore, incorporating SEL concepts requires comprehensive training to ensure that staff are sensitive to how cultural experiences impact how youth interact with the content. Ignoring the lived experience of youth who experience racism or discrimination can lead to a disconnect between SEL content and youths’ self-identity, making the curriculum ineffective [31]. Programs may either lack the additional time needed to incorporate SEL skills or are using an SEL curriculum that does not align with their programs, which leads to inconsistent use of the curriculum. To mitigate these barriers, the EMPOWER SEL curriculum has been co-developed with after-school partners and informed by implementation science factors, such as the Consolidated Framework for Implementation Research (CFIR) [30] to ensure organizational fit and the likeliness of its success.

## The pilot study: Objectives and study design

**Fig 1:**
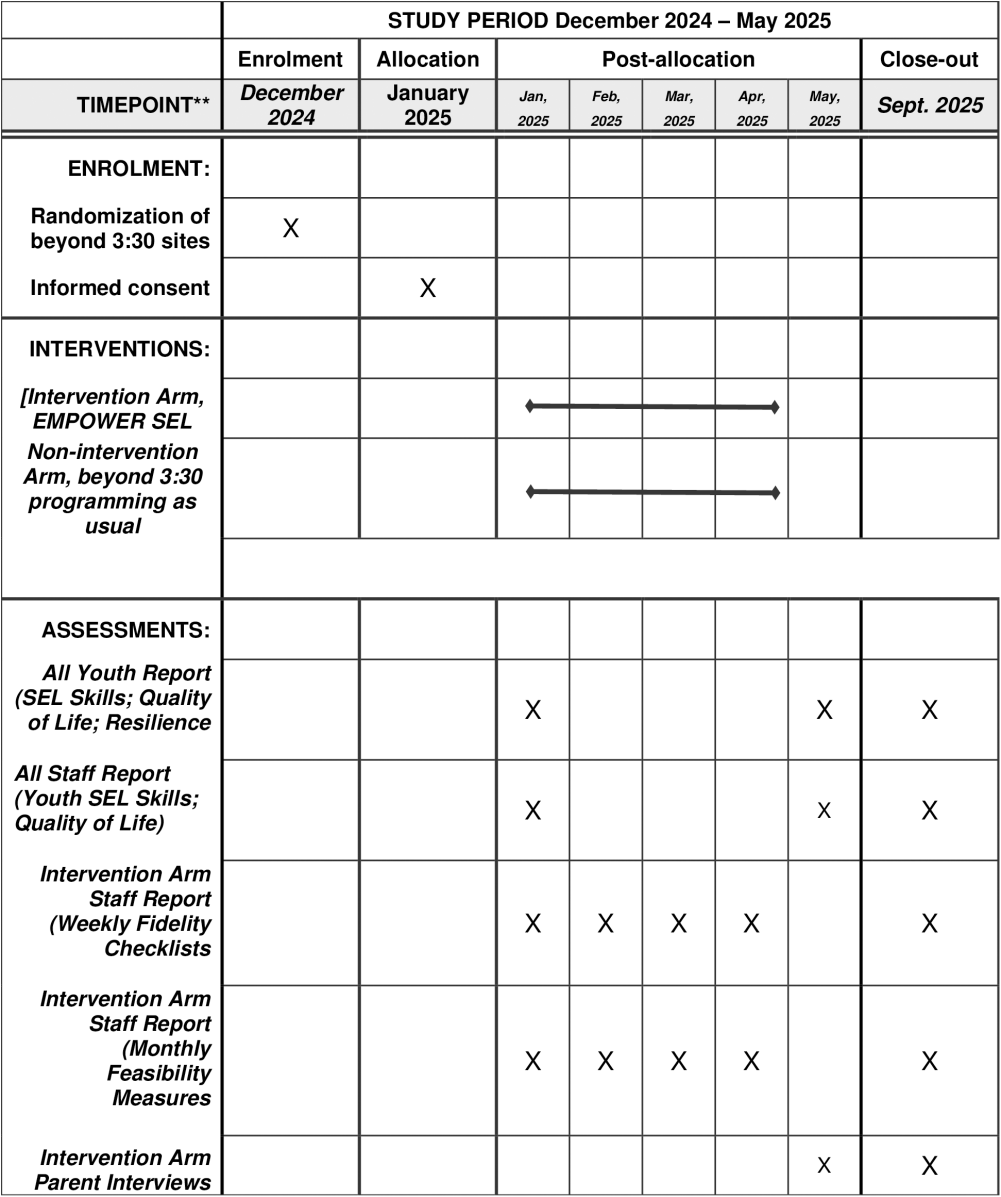
SPIRIT - Schedule of enrollment, interventions, and assessments.

This study aims to assess the feasibility and preliminary efficacy of the novel SEL curriculum with one after-school program partner that operates out of 20 sites, and serves approximately 600 youth in grades 6-8. Fig 1 details the SPIRIT schedule of enrollment. There are two co-primary objectives, the first is to test the feasibility, acceptability, and appropriateness of the EMPOWER SEL curriculum, and, the second is to test the preliminary efficacy of the EMPOWER SEL curriculum. The secondary objective is to test whether there are changes to youth’s overall functioning and resilience as a result of participating in the EMPOWER SEL intervention. Testing curricula using implementation science principles such as feasibility, fidelity, and implementation quality [30,32,33] is a best practice to share “what a program[] looks like in reality compared to what a program[] is conceived to be in theory” [33] p334). Numerous studies illustrate adherence to principles of implementation science in their monitoring of feasibility of SEL programs delivered in school [34,35] and after-school settings [36,37]. The outcomes we have chosen to assess feasibility, acceptability, and appropriateness are informed by the CFIR framework [30], the effectiveness-implementation hybrid trial design typology [38], as well as guidelines to inform the design of pilot and feasibility studies [39–41] and SEL program implementation [33]. The constructs we will assess have been organized into three overarching constructs that include feasibility, acceptability, and appropriateness.

This study design is a covariate-constrained randomized controlled trial with 10 sites assigned to the intervention arm, and 10 sites assigned to the no-intervention control arm. The no-intervention control arm was chosen as a comparator as there is already informal SEL programming that is delivered at the after-school program, and we wanted to compare the effects of a formalized program. This design will allow us to discuss the co-primary objective of the efficacy of the EMPOWER SEL curriculum. Evaluating the outcomes for youth who participate in SEL interventions is critical in understanding program effects. Using a randomized controlled study design is considered a “gold standard” in clinical research as it allows for causal explanations of the effects of interventions [42]. The outcomes to inform the secondary objectives are overall functioning and resilience.

## Methods

### Procedure

This trial was accepted by the research ethics board (REB#2024/111) at the academic mental health hospital on November 26^th^, 2024 and was registered with clinicaltrials.gov ID: NCT06619639. The trial will be conducted within one after-school program partner that offers free programming in underserved communities, with approximately 600 youth in grades 6-8. The program’s core focus is on sports and recreation activities, nutrition and cooking, and math and homework support in a safe and inclusive environment. The program aims to assist youth to succeed academically and aims to embed skills to help them flourish in and outside of school. All 20 sites, and their respective program staff, youth and parents/caregivers are eligible for participation in the trial. Program staff, youth, and parents/caregivers of youth who attend the after-school program will be included in the trial if they: (1) willingly consent, (2) are program staff who work with youth between 11-14 years or are in grades 6-8, (3), are youth between 11-14 years or are in grades 6-8, or (4) are parents/caregivers with children who are 11-14 years, or are in grades 6-8. Program staff and youth in the intervention groups who do not consent to participate in research measures will still deliver and be part of the intervention through their involvement with the after-school program.

The intervention sites will deliver the EMPOWER SEL curriculum, a 16-week program that addresses the CASEL [13] core competencies. The curriculum follows a sequence with lesson plans in self-awareness taught first, self-management second, social awareness and relationship skills third, and decision-making fourth. There are four weekly lesson plan topics within each of the four domains, which comprise the 16-week program. Weekly lesson plan topics are integrated into existing program activities twice per week and informally brought up as many times as the program staff feel is appropriate (for example, program staff can reinforce youth learning when they hear youth using the knowledge/skills from the lesson plan topic outside of formal instruction). As part of the EMPOWER SEL intervention, program staff will receive a 5-hour training prior to implementation that covers content on SEL for facilitators, a critical part of SEL implementation is to build program staff SEL capacity [33], and specifics about the curriculum with an opportunity for practice. Weekly coaching will be provided throughout the 16-week intervention to support program staff with curriculum delivery, an important facet of implementation science to ensure quality and fidelity [33]. As part of their work as program staff leading the after-school program sites, they are trained and responsible for managing negative and positive youth reactions and responses within the program generally. Thus, should youth have a negative response to the SEL lesson plans program staff will pivot their approach to ensure youth’s safety. Program staff work in a team-setting with a site supervisor to collaborate with. This is an established part of the after-school program structure.

### Randomization

The 20 after-school program sites will be randomized into intervention (n = 10) and no-intervention control groups (n = 10). A covariate-constrained randomized with no-intervention control study design will be used to assess the co-primary objectives of feasibility and efficacy, and secondary objectives of the EMPOWER SEL curriculum. Ten sites will be assigned to the active intervention arm and 10 to the no-intervention control arm. Study sites will be randomized 1:1 to receive either the active intervention or no-intervention, and will continue with their usual programming. Covariate-constrained randomization [43] will be used to balance treatment allocation across two site-level characteristics: (1) anticipated class size (ordinal measure), and (2) facilitators who were part of an iterative trial to test procedures in May 2024 (binary measure). Given the relatively small number of sites and 2-site level characteristics, standard stratified randomization is not appropriate to balance sites as it may lead to incomplete strata [44]. Briefly, since the site-level covariates will be known prior to the start of the study, we will calculate the covariate imbalance in the two site-level characteristics for all possible treatment allocations of the 20 sites. We then select a subset of treatment allocations that minimizes the covariate imbalance. In order to maintain randomness, the actual treatment allocation will be selected from this selected subset. The covariate-constrained randomization process will be implemented in R using the method [45].

### Consent procedure

All participants, youth, program staff, and parents/caregivers, will be asked to consent using the REDCap e-Consent framework developed by the research mental health hospital partner. With evidence from Hein et al. [46] who assessed the optimal cut-off ages for young people to consent to medical decision making, which was 10.4 years, we decided to have youth consent rather than assent to participate in research. Youth, program staff, and parents/caregivers who are invited to participate in the study will be asked to provide Informed Consent prior to completing any research measures. During the review of the Consent Form, trained senior lab members will explain the purpose of the research study, the treatment intervention, the risks and benefits to participating, the expected duration of the subject’s participation, the subject’s responsibilities, the compensation for participating, confidentiality and privacy, and that all participation is entirely voluntary. The individual obtaining consent must also indicate that if participants do choose to withdraw, it will in no way affect their relationship with the after-school program with which they work at (program staff) or attend (youth, parents/caregivers). All potential participants will be asked to provide consent (1) that they have been informed of the purpose of research; (2) to participate in the study; (3) to be contacted for future research; and, (4) to their de-identified data being used for other research.

### Data collection

To assess our co-primary outcome of feasibility, program staff in the intervention groups will be asked to complete measures over the course of 16-weeks to assess feasibility. This includes having one program staff from each intervention site complete the fidelity measure, which is a researcher-created questionnaire that contains 7 open- and closed-ended items measuring program adherence, adaptation, dosage, and engagement [30,33,40]. After program staff conclude each SEL domain, they will also be asked to complete 12-items on a 5-point Likert-type scale that measure the composite scores, 4-items each for the *Acceptability of Intervention, Feasibility of Intervention*, and *Intervention Appropriateness* [47]. Youth in the active intervention arm will also be asked to complete an adapted *Acceptability of Intervention* [47] measure (4 items, 5-point Likert-type scale) in the post-intervention data collection. These feasibility measures show strong reliability for the acceptability of intervention (α=.83), feasibility of intervention (α=.88), and intervention appropriateness (α=.87) scales [47]. Measures of fidelity and feasibility will begin in January 2025 and be completed in April 2025.

The second co-primary outcome to assess the preliminary efficacy of the EMPOWER SEL program and the secondary outcome to examine whether youth’s overall functioning and resilience skills will be tested via baseline and post-intervention data from staff and youth across all intervention and no-intervention arms. SEL skills are critical in supporting mental well-being, as such they are associated with youth’s ability to cope in the face of challenges (resilience) as well as to their overall functioning [48]. The *Social Skills Improvement System – Social Emotional Learning* (SSIS-SEL Brief Scale) is a 20-item, Likert-type scale that has four questions for each of the following five sub-scales: self-awareness, self-management, social awareness, relationship skills, and decision-making skills. Although there is strong reliability in the scales development, α=.79 to α=.87 across the subscales for the teacher version [49], and α=.67 to α=.72 for the youth version [50], it is suggested that the composite SEL skills score be used as both teacher (α=.93) and youth (α=.91) versions had a stronger composite SEL alpha scores than the subscales [51]. For scale validity, staff are required to know youth who they will report on for at least 4 weeks [52], a requirement that will be met as data collection will take place several months after the program commences.

Measures of overall functioning and resilience will also be completed at baseline and post-intervention to assess the secondary objective. The KIDSCREEN -10 [53] measures global quality of health scale in 10-items. The scale was developed to be completed by parents and youth with strong reliability scores for the initial 10-item parent measure (α=.78) and (α=.82 for the youth self-report version [54]. We adapted the parent scale to the perspective of after-school program staff. The parent scale frames questions from the parent’s perspective, for example, “has your child felt physically fit and well”. We have adapted this language to align with program staff perspectives, for example, “has the youth felt physically fit and well”? Only youth will be asked to complete the Connor-Davidson resilience measure (CD-RISC-10), which contains 10-items that measure a hardiness (e.g., flexibility, self-efficacy, emotion regulation, optimism, cognitive focus during stress). Items are scored on a 5-point Likert-type scale and added together for a total resilience score. Cronbach alpha was strong (α=.85) in the initial validation study [55]. Baseline data collection for the co-primary and secondary objectives will begin in December 2024 and post-intervention data will be collected in April 2025.

Finally, parents in the intervention groups will be asked to participate in a short interview to provide feedback about their child’s learning in the EMPOWER SEL program in April 2025. The following three questions guide the interview: (1) What are your overall thoughts about the inclusion of the SEL curriculum in [the after-school program]?; (2) Have you observed any changes in your child’s social-emotional competencies since your child began participating in the SEL curriculum?; and, (3) Is there anything else you’d like to share with us about the inclusion of the SEL curriculum at [the after-school program]?

**Fig 2:**
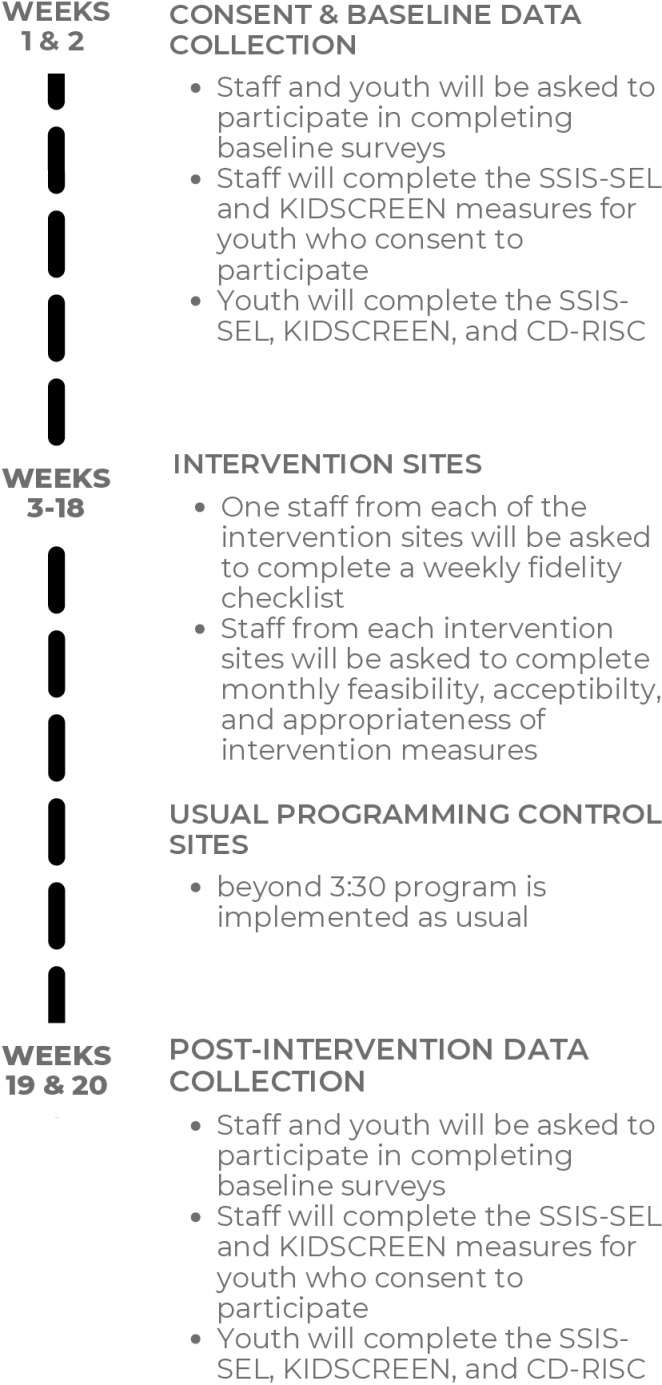
EMPOWER SEL pilot study timeline.

Fig 2 illustrates the timeline for the study, which is approximately 20-weeks in total. First, baseline data will be collected from consenting youth and program staff at all intervention and usual programming sites, after which the intervention sites will begin implementing the EMPOWER SEL curriculum and the no-intervention sites will continue with their regular programming. During implementation of the EMPOWER SEL, program staff who consent to complete study measures from each of the intervention sites will be asked to complete a weekly fidelity checklist, and monthly feasibility measures after each of the SEL domains have been completed. Once the 16-week EMPOWER SEL implementation is complete, all consenting youth and program staff at all intervention and no-intervention sites will be asked to complete the same measures of youth SEL skills, resilience, and overall functioning, as they did during the baseline data collection.

### Data management

All data for this trial will be entered and stored on the secure REDCap platform, which is housed on the central servers of the academic mental health hospital. The data on REDCap is backed up daily and is supported by the Research Informatics team at the academic mental health hospital. All participants will be assigned a study ID upon consenting to the study, such that names and identifying information are not tied to the data being entered on REDCap. Any questionnaires that are completed on paper will only contain a study ID and will be stored in a secure and locked office within the academic mental health hospital.

### Sample size & data analysis

Sample size and power calculations were performed using Power Analysis and Sample Size Software (PASS) [56]. Feasibility will be assessed by facilitators in the active intervention arm. We expect N = 20 program staff in total (10 active sites x 2 facilitators per site). A sample size of 20 program staff achieves an exact binomial 95% confidence interval width =  42.3% for the co-primary objective of feasibility endpoint 1, assuming the intervention is feasible for 14 out of 20 (70%) facilitators. The co-primary objective of efficacy of the EMPOWER SEL curriculum and the secondary objective of youth’s overall functioning and resilience will be assessed by program staff, youth, and parent/caregivers. A sample size of 20 clusters (10 active, 10 no-intervention control) with an average of 20 youth per cluster achieves 89% power (α=5%) to detect a small to medium effect size of 0.35 between arms in the secondary endpoint. We assume the coefficient of variation for the cluster sizes is 0.25, and the intracluster correlation (ICC) is 0.01.

Descriptive statistics will be used to analyze the fidelity and feasibility measures. Frequencies and proportions will summarize categorical measures; means, medians, standard deviations and ranges will summarize continuous measures. The frequency and proportion of program staff and youth who “completely agree” or “agree” that the SEL curriculum is feasible, acceptable, and appropriate will be calculated, along with exact binomial 95% confidence intervals. Authors (AB, BA, CM) constructed a traffic light approach with our quantitative data to report progression criteria. This strategy is informed by Mellor et al.’s [57] methodologic review of progression criteria. We have set our progression criteria threshold to 70% for feasibility measures. This means that 70% of program staff and youth “completely agree” or “agree” that the EMPOWER SEL curriculum is feasible, acceptable, and appropriate [47]. Scores in the green threshold point to acceptable levels of feasibility, acceptability, and appropriateness of the EMPOWER SEL program. Scores in the yellow (<60%) and red ( < 50%) threshold suggest that changes to the program may be needed. In assessments that yield yellow and red thresholds a consensus meeting with the EMPOWER collaborative will follow to determine necessary revisions and the best way to move forward. We will use IBM SPSS Statistics (Version 27) [58] to analyze this data using descriptive statistics.

The unit of analysis for the primary comparison for the SSIS-SEL brief scale is individual youth. Linear mixed models will be used to compare pre-post intervention SSIS-SEL scores between groups assuming the fixed effects of treatment group (EMPOWER vs. Usual Programming), time (0 and 18 weeks) post-initiation of intervention, and treatment-by-time interaction. To account for repeated measures, random effects of site and youth will be included in the model. A similar statistical approach will be conducted for the analysis of the secondary endpoints (overall functioning and resilience) between arms. The False Discovery Rate (ref) method will be used to adjust for multiple hypothesis tests of secondary endpoints. Analyses will adhere to the intention-to-treat principle [59]. Finally, parent interviews will be analyzed using an inductive thematic analysis approach [60] with NVivo software [28,61].

### Data monitoring

Site monitoring will occur throughout the trial to ensure the intervention is being implemented correctly and that all research measures are completed as intended. Program staff monitoring will occur via weekly virtual coaching sessions for the duration of the trial. Independent audits may be conducted by research staff to ensure monitoring practices are performed consistently across all participating sites. There data monitoring committee consists of the research team at the academic metnal health hospital (AB, MM, BA) who will be monitoring incoming weekly and monthly data. We do not anticipate any adverse events for youth, program staff, or parents who participate in completing study measures. Any adverse events will be addressed on a site-by-site basis. After-school program staff are already trained to identify youth distress within the context of the program and will monitor youth safety and will work to minimize experiences of distress during youth involvement in the SEL curriculum. Program staff will inform their administrators as well as the research team should any youth experience distress during the SEL curriculum implementation. Resources for mental health support will be shared with the youth participant and their parent/caregiver.

### Dissemination policy

There are no publication restrictions on the findings of this trial. We intend to share the findings with the after-school program community via newsletters, and share this project more broadly with community and academic audiences via peer-reviewed journals and conferences.

## Supporting information

S1 File REB Protocol - EMPOWER –NoLogo. (DOCX)

S2 File SPIRIT-Outcomes-2022-Checklist-with-SPIRIT-2013. (PDF)

## References

[1] Castelpietra G, Knudsen A, Agardh E, Armocida B, Beghi M, Iburg K. The burden of mental disorders, substance use disorders and self-harm among young people in Europe, 1990-2019: findings from the Global Burden of Disease Study 2019. Lancet Reg Health-Eur. 2022;1(16):100341. 10.1016/j.lanepe.2022.100341 PMC8980870 35392452

[2] Polanczyk GV, Salum GA, Sugaya LS, Caye A, Rohde LA. Annual research review: a meta-analysis of the worldwide prevalence of mental disorders in children and adolescents. J Child Psychol Psychiatry. 2015;56(3):345–65. doi: 10.1111/jcpp.12381 25649325

[3] Malla A, Shah J, Iyer S, Boksa P, Joober R, Andersson N, et al. Youth mental health should be a top priority for health care in Canada. Can J Psychiatry. 2018;63(4):216–22. doi: 10.1177/0706743718758968 29528719 PMC5894919

[4] Solmi M, Radua J, Olivola M, Croce E, Soardo L, Salazar de Pablo G, et al. Age at onset of mental disorders worldwide: large-scale meta-analysis of 192 epidemiological studies. Molecular Psychiatry. 2022;27:281–95. 34079068 10.1038/s41380-021-01161-7 PMC8960395

[5] Racine N, McArthur BA, Cooke JE, Eirich R, Zhu J, Madigan S. Global prevalence of depressive and anxiety symptoms in children and adolescents during COVID-19: a meta-analysis. JAMA Pediatr. 2021;175(11):1142–50. doi: 10.1001/jamapediatrics.2021.2482 34369987 PMC8353576

[6] Parenteau AM, Boyer CJ, Campos LJ, Carranza AF, Deer LK, Hartman DT, et al. A review of mental health disparities during COVID-19: evidence, mechanisms, and policy recommendations for promoting societal resilience. Dev Psychopathol. 2023;35(4):1821–42. doi: 10.1017/S0954579422000499 36097815 PMC10008755

[7] Fante-Coleman T, Jackson-Best F. Barriers and facilitators to accessing mental healthcare in Canada for black youth: a scoping review. Adolescent Res Rev. 2020;5(2):115–36. doi: 10.1007/s40894-020-00133-2

[8] Raphael D, Byrant T, Mikkonen J, Raphael A. Social determinants of health: the Canadian facts. 2020. Available from: http://www.thecanadianfacts.org/.

[9] Atfab A, Druss B. Addressing the mental health crisis in youth—sick individuals or sick societies? J Am Med Assoc. 2023;80(9):2. 10.1001/jamapsychiatry.2023.1298 37342020

[10] Gajaria A, Guzder J, Rasasingham R. What’s race got to do with it? A proposed framework to address racism’s impacts on child and adolescent mental health in Canada. J Can Acad Child Adolesc Psychiatry. 2021;30(2):131–7. 33953765 PMC8056965

[11] Durlak JA, Mahoney JL, Boyle AE. What we know, and what we need to find out about universal, school-based social and emotional learning programs for children and adolescents: a review of meta-analyses and directions for future research. Psychol Bull. 2022;148(11–12):765–82. doi: 10.1037/bul0000383

[12] Miyamoto K, Huerta MC, Kubacka K. Fostering social and emotional skills for well‐being and social progress. Euro J Educ. 2015;50(2):147–59. doi: 10.1111/ejed.12118

[13] The Collaborative of Academic, Social and Emotional Learning. What is the CASEL framework? 2024b. Available from: https://casel.org/fundamentals-of-sel/what-is-the-casel-framework/.

[14] Moore B, Gregory R. Decision making as a pedagogy for social emotional learning. Soc Emot Learn: Res Pract Policy. 2024;3:100034. doi: 10.1016/j.sel.2024.100034

[15] Knowledge Institute on Child and Youth Mental Health and Addictions CYMHA. Stemming the tide: Investing early in the mental health of Ontario’s 7- to 12-year-olds. Policy Paper. 2022.

[16] Masten A. Resilience from a developmental systems perspective. World Psychiatry. 2019;18(1):2. 10.1002/wps.20591 PMC6313232 30600628

[17] Durlak JA, Weissberg RP, Pachan M. A meta-analysis of after-school programs that seek to promote personal and social skills in children and adolescents. Am J Community Psychol. 2010;45(3–4):294–309. doi: 10.1007/s10464-010-9300-6 20300825

[18] Taylor RD, Oberle E, Durlak JA, Weissberg RP. Promoting positive youth development through school-based social and emotional learning interventions: a meta-analysis of follow-up effects. Child Dev. 2017;88(4):1156–71. doi: 10.1111/cdev.12864 28685826

[19] van de Sande MCE, Fekkes M, Kocken PL, Diekstra RFW, Reis R, Gravesteijn C. Do universal social and emotional learning programs for secondary school students enhance the competencies they address? A systematic review. Psychol Sch. 2019;56(10):1545–67. doi: 10.1002/pits.22307

[20] Cipriano C, Strambler MJ, Naples LH, Ha C, Kirk M, Wood M, et al. The state of evidence for social and emotional learning: a contemporary meta-analysis of universal school-based SEL interventions. Child Dev. 2023;94(5):1181–204. doi: 10.1111/cdev.13968 37448158

[21] Schonert-Reichl KA. Advancements in the landscape of social and emotional learning and emerging topics on the horizon. Educ Psychol. 2019;54(3):222–32. doi: 10.1080/00461520.2019.1633925

[22] Frazier SL, Cappella E, Atkins MS. Linking mental health and after school systems for children in urban poverty: preventing problems, promoting possibilities. Adm Policy Ment Health. 2007;34(4):389–99. doi: 10.1007/s10488-007-0118-y 17340183

[23] Shim RS, Compton MT. Addressing the social determinants of mental health: if not now, when? if not us, who? Psychiatr Serv. 2018;69(8):844–6. doi: 10.1176/appi.ps.201800060 29852822

[24] Compton MT, Shim RS. Mental illness prevention and mental health promotion: when, who, and how. Psychiatr Serv. 2020;71(9):981–3. doi: 10.1176/appi.ps.201900374 32867610

[25] Balter A, Racine N, Al-Khooly D, Somir I, Bandoles E, Utchay C, et al. Strengthening youth emotional and behavioral well-being through community–academic partnership: the EMPOWER Project. Health Promot Pract. 2024. 10.1177/15248399241255372 39066621

[26] Balter A, Sibalus A, Moloney M, Suri A, Racine N, Pierre S. The development of the EMPOWER social-emotional learning curriculum for youth in grades 6-8.

[27] Balter A, Pulat D, Suri A, Moloney M, Al-Khooly D, Somir I. Finding a needle in a haystack: A systematic approach for searching through public databases for youth mental well-being programs. J Sch Health. 2025. doi: 10.1111/josh.13536 39757132

[28] Brodkin S, Sibalis A, Bedard AV, Deluca A, Suri A, Balter A, et al. Mental health needs assessment for youth in out of school programs: a scoping review. J Commun Appl Soc Psychol. 2024;34(5):e2873. doi: 10.1002/casp.2873

[29] Knight MA, Haboush-Deloye A, Goldberg PM, Grob K. Strategies and tools to embrace prevention with upstream programs: a novel pilot program for enhancing social and emotional protective factors in middle school students. Child Sch. 2019;41(4):213–20. doi: 10.1093/cs/cdz020

[30] Damschroder LJ, Aron DC, Keith RE, Kirsh SR, Alexander JA, Lowery JC. Fostering implementation of health services research findings into practice: a consolidated framework for advancing implementation science. Implement Sci. 2009;4:50. doi: 10.1186/1748-5908-4-50 19664226 PMC2736161

[31] Jones S, Brush K, Ramirez T, Mao Z, Marenus M, Wettje S, et al. Navigating SEL from the inside out, looking inside & across 33 leading SEL programs: a practical resource for schools and OST providers. Preschool & Elementary Focus. The EASEL Lab The Harvard Graduate School of Education with Funding from the Wallace Foundation. 2021.

[32] Carroll C, Patterson M, Wood S, Booth A, Rick J, Balain S. A conceptual framework for implementation fidelity. Implement Sci. 2007;2:40. doi: 10.1186/1748-5908-2-40 18053122 PMC2213686

[33] Durlak JA. Programme implementation in social and emotional learning: basic issues and research findings. Camb J Educ. 2016;46(3):333–45. doi: 10.1080/0305764x.2016.1142504

[34] Dowling K, Barry MM. Evaluating the implementation quality of a social and emotional learning program: a mixed methods approach. Int J Environ Res Public Health. 2020;17(9):3249. doi: 10.3390/ijerph17093249 32392698 PMC7246810

[35] Green AL, Ferrante S, Boaz TL, Kutash K, Wheeldon‐Reece B. Social and emotional learning during early adolescence: effectiveness of a classroom‐based SEL program for middle school students. Psychol Sch. 2021;58(6):1056–69. doi: 10.1002/pits.22487

[36] Arlinghaus K, Bell A, Goodman L, Sherwood N, McMorris B. DiscoverU: A feasibility study of an afterschool mentoring program for adolescents that integrates social emotional learning, physical activity, and mindful eating. J Clin Transl Sci. 2023.

[37] Olive C, McCullick BA, Tomporowski P, Gaudreault KL, Simonton K. Effects of an after-school program focused on physical activity and social–emotional learning. J Youth Dev. 2020;15(6):292–305. doi: 10.5195/jyd.2020.889

[38] Curran GM, Bauer M, Mittman B, Pyne JM, Stetler C. Effectiveness-implementation hybrid designs: combining elements of clinical effectiveness and implementation research to enhance public health impact. Med Care. 2012;50(3):217–26. doi: 10.1097/MLR.0b013e3182408812 22310560 PMC3731143

[39] Bowen DJ, Kreuter M, Spring B, Cofta-Woerpel L, Linnan L, Weiner D, et al. How we design feasibility studies. Am J Prev Med. 2009;36(5):452–7. doi: 10.1016/j.amepre.2009.02.002 19362699 PMC2859314

[40] Pearson N, Naylor P-J, Ashe MC, Fernandez M, Yoong SL, Wolfenden L. Guidance for conducting feasibility and pilot studies for implementation trials. Pilot Feasibility Stud. 2020;6(1):167. doi: 10.1186/s40814-020-00634-w 33292770 PMC7603668

[41] Teresi JA, Yu X, Stewart AL, Hays RD. Guidelines for designing and evaluating feasibility pilot studies. Med Care. 2022;60(1):95–103. doi: 10.1097/MLR.0000000000001664 34812790 PMC8849521

[42] Zabor EC, Kaizer AM, Hobbs BP. Randomized controlled trials. Chest. 2020;158(1S):S79–87. doi: 10.1016/j.chest.2020.03.013 32658656 PMC8176647

[43] Moulton LH. Covariate-based constrained randomization of group-randomized trials. Clin Trials. 2004;1(3):297–305. doi: 10.1191/1740774504cn024oa 16279255

[44] Ivers NM, Halperin IJ, Barnsley J, Grimshaw JM, Shah BR, Tu K, et al. Allocation techniques for balance at baseline in cluster randomized trials: a methodological review. Trials. 2012;13:120. doi: 10.1186/1745-6215-13-120 22853820 PMC3503622

[45] Carter BR, Hood K. Balance algorithm for cluster randomized trials. BMC Med Res Methodol. 2008;8:65. doi: 10.1186/1471-2288-8-65 18844993 PMC2588445

[46] Hein IM, Troost PW, Lindeboom R, Benninga MA, Zwaan CM, van Goudoever JB, et al. Accuracy of the MacArthur competence assessment tool for clinical research (MacCAT-CR) for measuring children’s competence to consent to clinical research. JAMA Pediatr. 2014;168(12):1147–53. doi: 10.1001/jamapediatrics.2014.1694 25317644

[47] Weiner BJ, Lewis CC, Stanick C, Powell BJ, Dorsey CN, Clary AS, et al. Psychometric assessment of three newly developed implementation outcome measures. Implement Sci. 2017;12(1):108. doi: 10.1186/s13012-017-0635-3 28851459 PMC5576104

[48] Ungar M, Jefferies P. Becoming more rugged and better resourced: the r2 resilience program’s© psychosocial approach to thriving. Front Psychol. 2021;12:745283. doi: 10.3389/fpsyg.2021.745283 34955964 PMC8702437

[49] Anthony CJ, Elliott SN, DiPerna JC, Lei P. Initial development and validation of the social skills improvement system—social and emotional learning brief scales-teacher form. J Psychoeduc Assess. 2020;39(2):166–81. doi: 10.1177/0734282920953240

[50] Anthony CJ, Elliott SN, DiPerna JC, Lei P-W. The SSIS SEL Brief Scales-student form: initial development and validation. Sch Psychol. 2020;35(4):277–83. doi: 10.1037/spq0000390 32673055

[51] Anthony CJ, Brann KL, Elliott SN, Garis EJ. Examining the structural validity of the SSIS SEL brief scales—Teacher and student forms. Psychol Sch. 2021;59(2):260–80. doi: 10.1002/pits.22607

[52] Elliott S, Anthony C, DiPerna J, Lei P-W, Gresham F. SSIS™ SEL brief mental health scales expanded user guide & technical manual multi-informant assessments of students’ social emotional learning competencies and emotional behavior concerns. 2021.

[53] Child Public Health. KIDSCREEN-10. 2024.

[54] Ravens-Sieberer U, Erhart M, Rajmil L, Herdman M, Auquier P, Bruil J, et al. Reliability, construct and criterion validity of the KIDSCREEN-10 score: a short measure for children and adolescents’ well-being and health-related quality of life. Qual Life Res. 2010;19(10):1487–500. doi: 10.1007/s11136-010-9706-5 20668950 PMC2977059

[55] Campbell-Sills L, Stein MB. Psychometric analysis and refinement of the Connor-Davidson Resilience Scale (CD-RISC): validation of a 10-item measure of resilience. J Trauma Stress. 2007;20(6):1019–28. doi: 10.1002/jts.20271 18157881

[56] PASS. Power analysis and sample size software. Kaysville, Utah, USA: NCSS, LLC.; 2024. Available from: www.ncss.com/software/pass.

[57] Mellor K, Eddy S, Peckham N, Bond CM, Campbell MJ, Lancaster GA, et al. Progression from external pilot to definitive randomised controlled trial: a methodological review of progression criteria reporting. BMJ Open. 2021;11(6):e048178. doi: 10.1136/bmjopen-2020-048178 34183348 PMC8240572

[58] IBM Corp. SPSS Statistics for Windows, Version 27.0. Armonk, NY: IBM Corp. 2020.

[59] Benjamini Y, Hochberg Y. Controlling the false discovery rate: a practical and powerful approach to multiple testing. J R Stat Soc. 1995;57(1):289–300. doi: 10.1111/j.2517-6161.1995.tb02031.x

[60] Braun V, Clarke V. Using thematic analysis in psychology. Qualit Res Psychol. 2006;3(2):77–101. doi: 10.1191/1478088706qp063oa

[61] Lumivero. NVivo, version 27. 2024. Available from: www.lumivero.com

