# Supplementary material for: A protocol of a randomized control trial to test the feasibility and efficacy of the EMPOWER social-emotional learning curriculum for youth aged 11–14 years in after-school settings
Source: PLoS One. 2025 Mar 17;20(3):e0319398. doi: 10.1371/journal.pone.0319398 (PMC11913269; doi:10.1371/journal.pone.0319398)
Supplement: S1 File — (DOCX) [file pone.0319398.s001.docx]

**Cluster randomized trial of a novel social-emotional learning curriculum in an after-school program for youth, 11-14 years**

**Protocol Identifying Number: (**2024/111**)**

**Principal Investigator: Dr. Brendan Andrade**

**Version Number: 5.0**

**Version Date: *<18-11-2024>***

| **Sponsor:**  **Principal Investigator:**  **Qualified Investigator (if different from the PI):** | *Centre for Addiction and Mental Health*  *The Margaret and Wallace McCain Centre for Child, Youth and Family Mental Health*  *80 Workman Way, Toronto, Ontario*  *Dr. Brendan Andrade*  *80 Workman Way - 1109*  *x. 33642*  *Same as PI* |
| --- | --- |
| **Funder:** | *Les Lois Shaw Foundation*  *3109 Bloor St West*  *Toronto, ON* |

**Table of Contents**

STATEMENT OF COMPLIANCE 5

LIST OF ABBREVIATIONS 6

CLINICAL TRIAL SUMMARY 7

1.0 INTRODUCTION 8

1.1 Background 8

1.2 Study Intervention 8

1.3 Preclinical Data to Date 8

1.4 Clinical Data to Date 8

1.5 Risks/Benefits 8

2.0 CLINICAL TRIAL OBJECTIVES 8

2.1 Primary Objectives 8

2.2 Secondary Objectives 9

3.0 CLINICAL TRIAL DESIGN 9

3.1 Overall Design 9

3.2 Primary Endpoints 9

3.3 Secondary Endpoints 9

4.0 PARTICIPANT SELECTION, RECRUITMENT AND WITHDRAWAL 10

4.1 Target Population 10

4.2 Participant Recruitment and Screening 10

4.3 Equity, Diversity and Inclusion Considerations 11

4.4 Eligibility Criteria 12

4.4.1 Inclusion Criteria 12

4.4.2 Exclusion Criteria 12

4.5 Lifestyle Considerations 13

4.6 Screen Failures 13

4.7 Participant Withdrawal Criteria 14

4.7.1 When and How to Withdraw Participants 14

4.7.2 Follow-up for Withdrawn Participants 14

5.0 STUDY INTERVENTION 15

5.1 Description 15

5.2 Treatment Regimen 16

5.3 Method for Assigning Participants to Treatment Groups 16

5.4 Administration of Study Intervention 16

5.5 Participant Compliance Monitoring 16

5.6 Concomitant Therapy 16

5.7 Packaging 17

5.8 Blinding of Study Intervention 17

5.9 Receiving, Storage, Dispensing and Return 17

5.9.1 Receipt of Study Intervention Supplies 17

5.9.2 Storage 17

5.9.3 Dispensing of Study Intervention 17

5.9.4 Return or Destruction of Study Intervention 18

6.0 RESEARCH PROCEDURES 18

6.1 Research Visits 18

6.2 Schedule of Events 21

7.0 STATISTICAL PLAN 22

7.1 Sample Size Determination 22

7.2 Statistical Methods 22

8.0 SAFETY AND ADVERSE EVENTS 22

8.1 Definitions 22

8.2 Recording of Adverse Events 24

8.3 Reporting of Serious Adverse Events 24

8.3.1 Investigator Reporting: Notifying the Sponsor 24

8.3.2 Investigator Reporting: Notifying the REB 25

8.3.3 Sponsor Reporting of Adverse Events: Notifying Health Canada 25

8.3.4 Sponsor Reporting of Adverse Events: Notifying Sites 25

8.4 Reporting of Device Deficiencies 26

8.5 Safety Management Plan 26

8.6 Unblinding Procedures 26

8.7 Data and Safety Monitoring Board 27

9.0 CLINICAL TRIAL DISCONTINUATION AND CLOSURE 27

9.1 Clinical Trial Discontinuation 27

10.0 DATA HANDLING AND RECORD KEEPING 28

10.1 Source Documents & Case Report Forms 28

10.2 Study Equipment Calibration Records 28

10.3 Protocol Deviations 28

10.4 Record Retention 28

10.5 Clinical Trial Registration 29

11.0 STUDY MONITORING, AUDITING, AND INSPECTING 29

11.1 Study Monitoring Plan 29

11.2 Auditing and Inspecting 30

12.0 ETHICAL CONSIDERATIONS 30

12.1 Research Ethics Board (REB) Approval 30

12.2 Informed Consent Process & Documentation 31

13.0 PRIVACY AND CONFIDENTIALITY 33

14.0 CLINICAL TRIAL FINANCES 34

14.1 Funding Source 34

14.2 Conflict of Interest 34

15.0 PUBLICATION POLICY/DATA SHARING 34

15.1 Future Secondary Use of Data 35

16.0 REFERENCES 36

# STATEMENT OF COMPLIANCE

This pilot trial of a novel social-emotional learning curriculum will be carried out in accordance with the following:

- International Council for Harmonization Good Clinical Practice (ICH GCP)
- Tri-Council Policy Statement 2022 (TCPS 2)
- Personal Health Information Protection Act (PHIPA), 2004; Chapter 3 Schedule A (PHIPA) and applicable regulations
- Institutional and REB policies and procedures

________________________________ _____________________________

Signature of PI Date

*or*

Signature of site PI

# LIST OF ABBREVIATIONS

*AE* *Adverse Event*

*CRF*  *Case report form(s)*

*GCP*  *Good Clinical Practice*

*ICF*  *Informed consent form*

*PHI* *Personal Health Information*

*PHIPA* *Personal Health Information Protection Act*

*PI* *Principal Investigator*

*QI* *Qualified Investigator*

*SAE* *Serious Adverse Event*

*TCPS 2* *Tri-Council Policy Statement*

SEL *Social-Emotional Learning*

# CLINICAL TRIAL SUMMARY

| **Title** | Cluster-randomized trial of a novel social-emotional learning curriculum in an after-school program for youth, aged 11-14 years. |
| --- | --- |
| **Short Title** | Cluster-randomized trial of a novel social-emotional learning curriculum in an after-school setting. |
| **Phase** | Phase 1/2 |
| **Methodology** | Our first co-primary objective is to examine the feasibility of a novel social-emotional learning (SEL) curriculum, called EMPOWER SEL, within beyond 3:30, an after-school program that operates across 20 sites in the greater Toronto area. We will do this by asking after-school program staff in the active intervention arm to complete weekly online fidelity checklists for the duration of the program (16-weeks), complete 34 feasibility, acceptability, and appropriateness of intervention measures (Weiner et al., 2017) after each SEL domain is taught, and attend weekly coaching meetings to support the implementation of the EMPOWER SEL curriculum. Youth will also be asked to complete the acceptability of intervention measure (Weiner et al., 2017) post-intervention.  Our second co-primary objective is to evaluate the efficacy of the EMPOWER SEL curriculum. We will do this by asking youth and after-school program staff to complete SEL competency measures (SSIS-SEL Brief Scale; Elliot et al., 2020) during pre- and post-EMPOWER SEL curriculum data collection.  Our secondary objectives are to assess whether there are changes to youth’s overall functioning and resilience as a result of the EMPOWER SEL curriculum. We will assess this by asking youth to complete the KIDSCREEN-10 (Child Public Health, 2023) and CD-RISC-10 (Connor Davidson Resilience Scale, 2007) measures; and after-school program staff to complete the KIDSCREEN-10 (Child Public Health, 2023) pre- and post-EMPOWER SEL curriculum implementation.  We will conduct a cluster stratified randomized with usual programming control study within the beyond 3:30 after-school program to assess feasibility and efficacy of the EMPOWER SEL curriculum. We will use covariate constrained randomization (Moulton, 2004) to balance the sites based on the class size at each site and facilitators who were a part of the iterative trial in May 2024. There are 20 sites within the beyond 3:30 program, 10 sites will be assigned to the active intervention arm and 10 sites will be assigned to the usual programming control arm.  After the active intervention arm completes implementing the 16-week EMPOWER SEL curriculum, and both intervention and usual programming control arms complete the post-EMPOWER SEL program measures, the study will be completed.  Data collection procedure:   - Quantitative Data: - After-school program staff will be sent a secure link to their respective questionnaires that they will complete via REDCap. Facilitators at each site will complete survey measures about each youth’s social emotional skills and overall functioning before and after the curriculum is implemented for the youth who consent to be in the study. This de-identified data, which means there are no names included, is for the research team only. This data will be grouped and no individual data will be analyzed. The data will be used to compare whether there were changes to youth social emotional learning and overall functioning with program participation.   - Staff will receive a gift card of $30/hour honorarium for participating in each of the pre- and post-EMPOWER SEL curriculum implementation surveys.   - After-school program staff will be sent a secure link to complete the weekly fidelity checklist via REDCap. As an honorarium, there will be a monthly draw for 2 $50 gift cards.   - After-school program staff will be sent a secure link to complete the monthly feasibility measures via REDCap. As an honorarium, there will be a monthly draw for 2 $50 gift cards.   - Two staff from CAMH will attend each site, pre- and post-SEL curriculum data collection, to distribute tablets and read each survey question aloud to the group of youth as they complete their surveys via REDCap. Youth aged 11-14 years will consent to their participation in the study. A pizza party at both pre- and post-EMPOWER SEL curriculum implementation data points will be provided for all youth, regardless of whether they complete study surveys or not. - Qualitative Data:   - Interviews with parents/caregivers post-SEL curriculum implementation. A $15 gift card will be provided as an honorarium for parents/caregivers.   **Measures:**  Social Skills Improvement System – Social Emotional Learning (SSIS-SEL Brief Scale;Elliot et al., 2020) is a 20-item, 4-point Likert-type scale that measures self-awareness, self-management, social awareness, relationship skills, and decision making as a composite score of social-emotional competency. Staff in both active intervention and usual programming arms will complete the SSIS-SEL Brief Scale for each youth they work with (avg. 20 youth/site) pre- and post-EMPOWER SEL curriculum intervention. Youth in active intervention and usual programming arms will complete the SSIS-SEL Brief Scale pre- and post-EMPOWER SEL curriculum intervention.  KIDSCREEN 10 (Child Public Health, 2023) is a 10-item, 5-point Likert-type scale that measures global quality of life. Staff in active intervention and usual programming arms will complete an adapted version of the KIDSCREEN for each youth they work with (avg. 20 youth/site) pre- and post-SEL curriculum intervention. Youth in active intervention and usual programming arms will complete the KIDSCREEN pre- and post-SEL curriculum intervention.  CD-RISC-10 (2007) is a 10-item, 5-point Likert-type scale that measures overall resilience. Youth in active intervention and usual programming arms will complete this measure pre- and post-SEL curriculum intervention.  Acceptability of Intervention Measure (Weiner et al., 2017) is a 4-item, 5-point Likert-type scale that measures a composite acceptability of intervention score. Staff in the active intervention arm will complete this measure every 4 weeks, after each social-emotional domain (self-awareness, self-management, social awareness and relationship skills, decision-making) is taught. Youth in the active intervention arm will complete this measure post-SEL curriculum implementation.  Feasibility of Intervention (Weiner et al., 2017) is a 4-item, 5-point Likert-type scale that measures a composite feasibility of intervention score. Staff in the active intervention arm will complete this measure every 4 weeks, after each social-emotional domain (self-awareness, self-management, social awareness and relationship skills, decision-making) is taught.  Intervention Appropriateness (Weiner et al., 2017) is a 4-item, 5-point Likert-type scale that measures a composite appropriateness of intervention score. Staff in the active intervention arm will complete this measure every 4 weeks, after each social-emotional domain (self-awareness, self-management, social awareness and relationship skills, decision-making) is taught.  One facilitator from each site in the active intervention arm will complete a weekly fidelity checklist that consists of 10 questions that assess adherence and adaptations to the lesson plan structure, facilitator perception of youth engagement, facilitator perception of youth’s generalization of SEL skills, and facilitator self-efficacy.  Notes documenting the weekly coaching sessions will not be systematically analyzed, rather they are taken to inform curriculum refinement and are a part of supporting implementing the SEL curriculum.  The following questions will guide the parent/caregiver interviews: *(1) What are your overall thoughts about the inclusion of the SEL curriculum in beyond 3:30? (2) Have you observed any changes in your child's social-emotional competencies since your child began participating in the SEL curriculum? (3) Is there anything else you'd like to share with us about the inclusion of the SEL curriculum at beyond 3:30?* One parent/caregiver from each site in the active intervention arm will be asked (non-probability convenience sample) to participate in a 30-minute (maximum) interview post-SEL curriculum implementation via Webex.  **Data collection time points:**  *2-weeks pre-EMPOWER SEL curriculum implementation*  - youth and facilitators in the active intervention and usual programming control arms will complete the measures for our second co-primary and secondary objectives.  *Weekly (once EMPOWER SEL curriculum begins)*  - facilitators in the active intervention arm will complete fidelity checklists via REDCap at the end of each week.  - facilitators in the active intervention arm will attend weekly coaching meetings at the end of each week to support the SEL curriculum implementation and collect qualitative information to refine the curriculum.  *Every 4 weeks (when facilitators complete an SEL domain)*  - facilitators in the active intervention arm will complete feasibility, acceptability, and intervention appropriate measures via REDCap every 4 weeks for 16 weeks. Feasibility measures are to be completed when facilitators complete the SEL domain they are teaching.  2*-weeks post-SEL curriculum*  - youth and facilitators in the active intervention and usual programming control arms will complete the measures for our second co-primary and secondary objectives via REDCap. Youth will be asked to complete an additional intervention appropriateness measure. Parents/caregivers in the active intervention arm will be asked to participate in a 30-minute interview. |
| **Clinical trial Duration** | The duration of this trial is approximately 20 -weeks (determined by when the pre-EMPOWER SEL curriculum data collection begins) which are broken down as follows:  1) We will collect pre-EMPOWER SEL curriculum data **2-weeks** before the curriculum is implemented from youth and facilitators in both the intervention and usual programming control arms. This will begin approximately mid-October 2024.  2) November 2024 - beginning of March 2025: the active intervention arm will implement the **16-week** EMPOWER SEL curriculum.  3) We will collect post-EMPOWER SEL curriculum data for**2-weeks** after the intervention arm completes implementing the SEL curriculum. |
| **Participating site(s)** | beyond 3:30 is an after-school program that operates across Toronto and hosts its programs in 20 schools (referred to as sites). |
| **Objectives** | The first co-primary objective is to examine the feasibility, acceptability, and appropriateness of our novel SEL curriculum within beyond 3:30.  The second co-primary objective is to evaluate the efficacy of the SEL curriculum.  The secondary objectives are to assess if there are changes to youth’s overall functioning and resilience as a result of the SEL curriculum. |
| **Number of Participants** | - Approximately 40 after-school program staff (20 in active control, 20 in usual programming control); - Approximately 600 youth aged 11-14 years (300 in active control, 300 in usual programming control); - Approximately 10-20 parents/caregivers whose children are in the active intervention arm. |
| **Study Intervention**  **Reference Therapy/Comparator** | Project EMPOWER’s SEL curriculum is a novel program developed by our community-academic partnership to reach youth aged 11-14 years attending the beyond 3:30 after-school program.  The curriculum addresses the SEL competencies outlined by The Collaborative of Academic, Social and Emotional Learning (CASEL; 2024), which include self-awareness, self-management, social awareness and relationship skills, and decision-making. One topic is formally embedded into beyond 3:30’s existing program activities twice per week. There are four topics within each SEL competency, and it takes 4-weeks to complete each domain. The entirety of the curriculum takes 16-weeks to implement.  The SEL curriculum is being compared to the beyond 3:30 program as usual without the curriculum (usual programming control arm). |
| **Duration of Intervention** | The intervention takes 16-weeks to implement, the study takes 20-weeks to complete. |
| **Statistical Methodology** | 1. Co-primary Objective: Feasibility 2. Acceptability, Feasibility, and Appropriateness of Intervention Measures   We’ve set our progression criteria threshold to 70% for feasibility measures. This means that 70% of facilitators and youth “completely agree” or “agree” that the EMPOWER SEL curriculum is feasible, acceptable, and appropriate. We use a traffic light approach (Mellor et al., 2021) where meeting the set threshold equates to a green light and that the curriculum can proceed into its next phases of testing. Changes to the SEL curriculum are required should the threshold not be met (50%-60% = yellow light, or under 50% = red light). In these cases, a consensus meeting with the EMPOWER collaborative (partners [beyond 3:30 and VOS] and staff, youth, and caregiver advisors) will follow to determine necessary revisions and determine the best way to move forward.   1. Fidelity checklists   Each of the constructs within the fidelity checklist are measured in one-item and are descriptive in nature. They will be analyzed as such.   1. Co-primary Objectives: Social-Emotional Learning 2. SSIS-SEL Brief Scale   The unit of analysis for the primary comparison is individual youth. Linear mixed models will be used to compare pre-post intervention SSIS-SEL scores between groups assuming the fixed effects of treatment group (EMPOWER vs. Usual Programming), time (0 and 18 weeks post-initiation of intervention, and treatment-by-time interaction. To account for repeated measures, random effects of site and youth will be included in the model.   1. Secondary Objectives (secondary) 2. KIDSCREEN and CD-RISC 10 Measures   A similar statistical approach as for co-primary endpoint 2 (SEL) will be used to compare secondary endpoints between arms.  **Sample size considerations**  Co-primary endpoint 1: Feasibility  Feasibility will be assessed by facilitators in the active intervention arm. We expect N=20 facilitators in total (10 active sites x 2 facilitators per site). A sample size of 20 facilitators achieves an exact binomial 95% confidence interval width = 42.3% for co-primary endpoint 1, assuming the intervention is feasible for 14 out of 20 (70%) facilitators.  Co-primary endpoint 2: Social emotional learning  A sample size of 20 clusters (10 active, 10 usual care) with an average of 20 youth per cluster achieves 89% power (α=5%) to detect a small to medium effect size of 0.35 between arms in the primary endpoint. We assume the coefficient of variation for the cluster sizes is assumed to be 0.25, and the intracluster correlation (ICC) is 0.01. |

# INTRODUCTION

## 1.1 Background

The prevalence and severity of youth mental health difficulties have risen dramatically over the past decade (Arakelyan et al., 2023; Polanczyk et al., 2015) and have been compounded by the COVID-19 pandemic (Kauhanen et al., 2023; Madigan et al., 2023). The pandemic highlighted existing health disparities among groups who face inequities in social determinants of health (Raphael et al., 2020). The same disparities exist for those who are facing mental health challenges, which are compounded due to intersectional factors including socio-economic status (Murthy, 2022), disability (Samji et al., 2022; Murthy, 2022), race (Fante-Coleman & Jackson-Best, 2020; Murthy, 2022), gender identity and sexual orientation (Samji et al., 2022; Murthy, 2022), and more.

Social emotional learning (SEL), under the evidence-based framework established by the Collaboration of Academic, Social and Emotional Learning (CASEL), refers to the process through which intrapersonal (e.g., self-awareness, self-management, decision making) and interpersonal competencies (e.g., social awareness, relationship skills, decision making) are taught and learned as necessary skills to navigate life stressors. SEL programs in school and after-school settings have multiplied in the past decade and are associated with beneficial outcomes for youth. These include improvements in (a) personal skills, such as self-regulation, responsible decision making self-perception, positive attitudes towards others; (b) social skills, such as prosocial behaviors (e.g., cooperation and helping others) and social awareness, including empathy, and (c) academic performance, as well as reductions in substance use, conduct problems, and emotional distress (Durlak et al., 2022; Taylor et al., 2017; Durlak et al., 2010). Research has demonstrated that increases in SEL competencies gained from school and after-school programming are sustained for anywhere between 56 weeks and 195 weeks (Taylor et al., 2017).

After-school programs can improve accessibility to mental well-being programming in communities. Given that after-school programs are often introduced to promote positive social activities outside the classroom, the integration of SEL concepts has been increasingly used in recent years (Durlak et al., 2022). After-school programs that emphasize the development of positive social and emotional skills have been shown to improve a variety of youth outcomes, such as increases in self-confidence, social behaviors, and academic outcomes (Durlak & Weissberg, 2010). These programs also seem to show a significant reduction in maladaptive behaviors, which may be critical in underserved communities where access to affordable mental health care is limited (Frazier et al., 2021; Durlak & Weissberg, 2010).

The EMPOWER project is a community-academic partnership between two after-school programs in Toronto (Visions of Science and Beyond 3:30) and researchers at CAMH. It was developed to enhance the mental well-being of youth ages 11-14 attending these programs in underserved and vulnerable neighbourhoods. As such, the study team has developed a novel SEL curriculum that can be integrated into community after-school programs, with hopes of increasing accessibility to mental wellness programming by improving key mental wellness outcomes for youth in these communities.

## 1.2 Study Intervention

The intervention being tested in this trial is a novel SEL curriculum for youth ages 11-14. The curriculum is designed to improve youth’s social emotional competencies and mental wellness through the integration of short SEL skills embedded into existing curriculum within after-school programs. The curriculum is based on CASEL’s five core SEL competencies; self-awareness, self-management, social awareness, relationship skills and decision-making. This curriculum takes 16 weeks to implement. There is an order to teach each domain (e.g., self-awareness, self-management, social awareness & relationship skills, decision-making) that includes 4 topics which are integrated each week. Each domain takes 4 weeks to complete. Each topic is taught with a brief 5–7-minute lesson plan that is integrated into one of the existing after-school program activities. Each weekly topic is introduced formally twice per week. The facilitator is also expected to informally mention or remind the youth of the topic throughout the week as many times as they see fit.

Facilitators are asked to embed the SEL lesson plans within an existing program activity to engage youth to build their social-emotional competencies in a way that doesn’t take away from their program culture or program activities. Although this curriculum takes a person-centered and trauma-informed approach, the focus is on SEL skills in the context of program activities (e.g. self-talk during a basketball game). With repeated exposure to the lesson plan topics each week, we anticipate that, over time, youth will begin to generalize and extend the SEL skills learned to other areas of their lives.

Beyond 3:30 has approved the implementation of this curriculum across all of its sites within the 2024/2025 school year. The intervention is not what participants are consenting to, rather, they are consenting to participating in completing study measures.

## 1.3 Preclinical Data to Date

We tested the initial feasibility of the lessons within the self-awareness domain at five beyond 3:30 sites in May 2024. We sought and were granted approval from QPER (#2022_005) to carry this out. Facilitators participated in a three-hour in-person training two weeks prior to the start of the iterative trial, which covered an overview of the EMPOWER project, the SEL curriculum structure, and the self-awareness domain lesson plans. Facilitators were asked to implement only the lessons within the self-awareness domain. This included teaching two self-awareness lesson plans per week (our initial curriculum included 8 lesson plans per domain, where facilitators taught two separate lesson plans each week) over four weeks. Facilitators participated in weekly thirty-minute coaching sessions with the study team to discuss any implementation successes and challenges. Upon the conclusion of testing the self-awareness domain, the study team engaged youth in thirty-minute focus groups at each of the sites and a one-hour focus group with facilitators to hear their perspectives on the initial implementation of the SEL curriculum.

Participant feedback from the iterative trial provided important insights into key aspects of the lesson plan concept and feasibility of integrating it into after-school settings. This feedback has resulted in changes that will inform the implementation for this pilot trial, where we will study the feasibility of the entire curriculum. Facilitators indicated on the post-training questionnaire that they felt confident and prepared to implement the curriculum after the initial training session; however, during the coaching sessions and in the post-intervention focus group, it was evident that more examples of SEL integration within program activities, as well as baseline SEL knowledge was needed for facilitators. As such, the facilitator training structure will be enhanced and broken into different segments for the pilot trial.

Facilitators indicated in the post-intervention data that overall, the curriculum was acceptable and appropriate for the youth in their programs, noting marginal improvements among some of the youth in terms of SEL competencies. Feasibility ratings for the intervention among facilitators were also quite high; however, the focus group revealed that more than half (5/8) of the facilitators felt as though the curriculum could be slowed down to accommodate youth who require more time to understand the SEL content, and to also account for the structure of the beyond 3:30 program where not all youth attend five days a week. The feedback regarding the pace of the curriculum resulted in changes to the curriculum structure, which now features one SEL topic per week instead of two.

The youth data provided insights into youth engagement strategies that are needed to successfully integrate the curriculum into the beyond 3:30 program. Multiple youth indicated that recreational activities (e.g. basketball, art) as opposed to more academic activities (e.g., journaling and writing) were preferable ways to learn about SEL skills. These results will inform the facilitator trainings.

## 1.4 Clinical Data to Date

There is no clinical data to date on the study intervention.

## 1.5 Risks/Benefits

**Risks:** Some of the questionnaires will ask youth and staff about topics that may be uncomfortable. As a result, these questionnaires could induce stress or trigger negative emotions. If youth participants become distressed, a research team member can assist and provide further resources by following the steps and consulting with the risk management standard operating procedures document (attached with this application). If specific questionnaires or questions make participants uncomfortable, they are free to skip them or indicate that they prefer not to answer. For staff participants who are not observed as they complete their surveys, information about voluntary participation and risk management are included in the consent form.

The control condition is necessary to evaluate the preliminary effectiveness of the intervention. However, all participants will receive the intervention by the end of the trial (the intervention arm will receive the full 16-week intervention. At the conclusion of this study (when post-EMPOWER SEL data is collected) the usual programming control arm will receive a shortened, approximately 12-week, intervention to align with the end of b3:30 programming for the school year.

**Benefits**: Participation in this study is important to increase our understanding of the feasibility and effectiveness of this novel SEL curriculum for youth at the beyond 3:30 program. As what we are seeking approval for is youth, staff, and caregiver participants to participate in data collection, it is unclear whether completing measures (youth and staff) or participating in an interview (caregivers) will result in direct benefits to participants. We hope the study findings will contribute to supporting and building youth’s SEL skills that may improve their and overall community mental wellness outcomes.

# CLINICAL TRIAL OBJECTIVES

## 2.1 Primary Objectives

Our first co-primary objective is to examine the feasibility of the EMPOWER SEL curriculum within the beyond 3:30 after-school program. We will do this by asking after-school program staff to complete weekly online fidelity checklists for the duration of the active intervention (16-weeks), and complete 4 feasibility, acceptability, and appropriateness of intervention measures (Weiner et al., 2017) for each domain that is taught (every 4 weeks). Youth will also be asked to complete the intervention appropriateness measure (Weiner et al., 2017) post-intervention.

Our second co-primary objective is to evaluate the efficacy of the EMPOWER SEL curriculum. We will do this by asking youth and after-school program staff to complete the SSIS-SEL Brief Scale (Elliot et al., 2020) during pre- and post-SEL curriculum implementation data collection.

## 2.2 Secondary Objectives

Our secondary objectives are to assess whether there are changes to youth’s quality of life and resilience as a result of the SEL curriculum. We will assess this by asking youth to complete the KIDSCREEN-10, CD-RISC-10 measures; after-school program staff to complete an adapted version of the KIDSCREEN-10; and caregivers to participate in a brief 30-minute interview post-SEL curriculum implementation.

# 3.0 CLINICAL TRIAL DESIGN

## 3.1 Overall Design

A cluster stratified randomized with usual programming control study will be used to assess the feasibility and efficacy of the EMPOWER SEL curriculum. There are 20 sites within the beyond 3:30 program. We will stratify the sites based on class size at each site, and facilitators who were a part of the iterative trial in May 2024. Ten sites will be assigned to the active intervention arm and 10 to the usual programming control arm.

**Trial Duration:** The duration of this trial is approximately 20-weeks and concludes once the post-EMPOWER SEL curriculum data is collected..

1) pre-SEL curriculum implementation data collection will begin approximately mid-October, 2024; youth and staff in the intervention and usual programming control arms will be asked to participate in the pre-SEL curriculum implementation data collection.

2) November 2024 - beginning of March 2025: the active intervention arm will implement the 16-week SEL curriculum. The usual programming control arm will proceed with the regular beyond 3:30 curriculum.

3) November 2024 – beginning of March 2025: facilitators in the active intervention arm will be asked to complete weekly fidelity checklists and monthly feasibility measures.

3) post-SEL curriculum data collection will begin approximately mid-March 2025; Youth and staff in the intervention and usual programming control arms will be asked to participate in the post-SEL curriculum implementation data collection. Caregivers will be asked to participate in a 30-minute interview.

**Duration of participant involvement in clinical trial:**

- Youth and staff in the active intervention arm will be involved in this study for approximately 20 weeks (mid-October 2024 – mid March 2025); 2-weeks pre-implementation data collection + 16-weeks to implement the SEL curriculum (and to complete feasibility and fidelity measures and attend weekly coaching sessions) + 2-weeks post-implementation data collection.
- Parents/caregivers in the active intervention arm only will be involved in this study for approximately 1-2 weeks at the conclusion of the SEL curriculum implementation.
- Youth and staff in the usual programming control arm will be involved in this study for approximately 20 weeks (mid-October 2024 – end of May 2025); 2-weeks pre-implementation data collection + 16-weeks of usual beyond 3:30 programming while the intervention arm delivers the SEL curriculum + 2-weeks post-implementation data collection.

**Expected clinical trial duration:** The total expected duration of the clinical trial is approximately 20 weeks.

**Data collection time points:**

*2-weeks pre-SEL curriculum implementation*

- youth and facilitators in both the active intervention and usual programming control arms will be asked to complete the measures for our second co-primary and secondary objectives.

*Weekly (once SEL curriculum begins)*

- facilitators in the active intervention arm be asked to will complete fidelity checklists via REDCap at the end of each week.

- facilitators in the active intervention arm will attend weekly coaching meetings at the end of each week to support the SEL curriculum implementation and provide an opportunity for us to collect qualitative information that will help refine the curriculum.

*Every 4 weeks (when facilitators complete an SEL domain)*

- facilitators in the active intervention arm will be asked to complete feasibility, acceptability, and intervention appropriate measures via REDCap every 4 weeks for 16 weeks. Feasibility measures are to be completed when facilitators complete the SEL domain they are teaching.

2*-weeks post-SEL curriculum*

- youth and facilitators in both the active intervention and usual programming control arms will be asked to complete the measures for our second co-primary and secondary objectives via REDCap. Youth will be asked to complete an additional intervention appropriateness measure. Parents/caregivers will be asked to participate in a 30-minute interview.

**Schematic Diagram:**


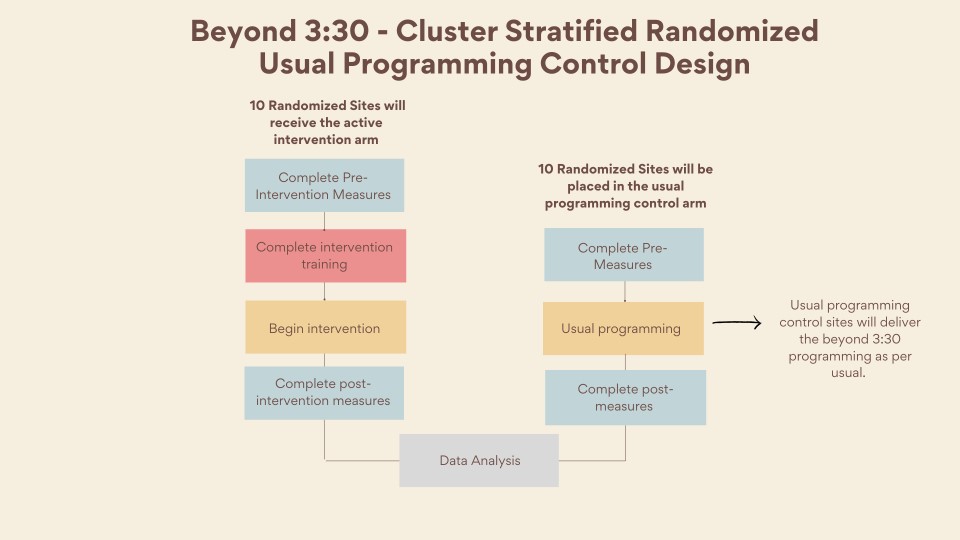


**Factors that may compromise the outcomes of the clinical trial:**

1. To ensure that we have representative groups in each the active intervention and usual programming control arms, we will stratify the sample of 20 sites.
   1. The class sizes amongst each site varies between 8-30 youth aged 11-14 years. We will stratify the class sizes at each site.
   2. We conducted an iterative trial of a segment (the self-awareness domain) of the SEL curriculum in May 2024 with 5 sites at beyond 3:30. We will stratify the facilitators who were a part of the iterative trial in May 2024 to reduce bias.
2. There may be facilitators from the active intervention arm who may engage in discussions and share information about the SEL curriculum with facilitators from the usual programming intervention arm. We will keep this possibility of bias in mind in our analysis and findings while comparing the active intervention and usual programming control arms.

## 3.2 Primary Endpoints

**Co-primary endpoint 1: Feasibility**

Feasibility will be assessed by facilitators in the active intervention arm. Facilitators will be asked to complete the *Acceptability of Intervention, Feasibility of Intervention,* and *Intervention Appropriateness* (Weiner et al., 2017) measures. Each of the three measures contains 4-items, are answered in 5-point Likert-type scale, and measure the composite score of the construct. Staff will be asked to complete these measures every 4 weeks, after each social-emotional domain (self-awareness, self-management, social awareness and relationship skills, decision-making) has been taught.

We’ve set our progression criteria threshold to 70% for feasibility measures. This means that 70% of facilitators and youth “completely agree” or “agree” that the SEL curriculum is feasible, acceptable, and appropriate. We use a traffic light approach (Mellor et al., 2021) where meeting the set threshold equates to a green light and that the curriculum can proceed into its next phases of testing. Changes to the SEL curriculum are required should the threshold not be met (50%-60% = yellow light, or under 50% = red light). In these cases, a consensus meeting with the EMPOWER collaborative (partners [beyond 3:30 and VOS] and staff, youth, and caregiver advisors) will follow to determine necessary revisions and determine the best way to move forward.

Additionally, one facilitator from each of the 10 active intervention sites will be asked to complete a weekly fidelity checklist that consists of 10 questions (researcher-created) that assesses adherence and adaptations to the lesson plan structure, facilitator perception of youth engagement, facilitator perception of youth’s generalization of SEL skills, and facilitator self-efficacy.

Youth in the active intervention arm will be asked to complete the 4 items in the Intervention Appropriateness (Weiner et al., 2017) measure post-SEL curriculum implementation.

**Co-primary endpoint 2: Social emotional learning skills**

Youth’s SEL skills will be assessed by youth themselves andfacilitators in both active intervention and usual prorgramming control arms, at the pre- and post-SEL curriculum implementation data collection time points. Facilitators and youth will be asked to complete the SSIS-SEL Brief (Elliot et al., 2020) measures which are each 20-items in length, answered on a 4-point Likert-type scale, and measure a composite score of social-emotional competency.

Parents/caregivers in the active intervention arm will be asked to participate in an interview post-SEL curriculum implementation.

## 3.3. Secondary Endpoints

Secondary efficacy endpoints include youth’s overall functioning and resilience as a result of the SEL curriculum. We will assess this by asking youth to complete the KIDSCREEN-10 (Child Public Health, 2023) and CD-RISC-10 (Connor Davidson Resilience Scale, 2007) measures; and after-school program staff to complete the KIDSCREEN-10 (Child Public Health, 2023) pre- and post-SEL curriculum implementation.

# 4 PARTICIPANT SELECTION, RECRUITMENT AND WITHDRAWAL

## 4.1 Target Population

Youth aged 11-14 years who attend the beyond 3:30 after-school program in Toronto, Ontario are the target population as are the staff of the program and caregivers/parents whose children attend the program. beyond 3:30, offers free after-school programming for youth between the ages of 8 and 14 in communities facing social and economic vulnerabilities, with a majority located in *emerging* and *neighbourhood improvement areas* (City of Toronto, 2024) across Toronto.

Beyond 3:30 has adopted the implementation of the SEL program into all of their sites as a new addition to their programming for the 2024/2025 school year. Therefore, there is no individual consent required to participate in the programming. What we engage in consent procedures with staff, youth, and caregivers is for participation in data collection.

Sites are classrooms, and beyond 3:30 divides its classroom by age—juniors (8-10 years) and seniors (11-14 years). There may be times when juniors participate in the SEL programming, which we as a partnership (CAMH and beyond 3:30) have determined is acceptable as the content with the SEL lesson plans are relevant and relatable for younger youth as well. Should this happen the juniors will receive and engage in the SEL content, but they will not be asked to participate in any data collection.

## 4.2 Participant Recruitment and Screening

Anticipated number of sites and participants to be enrolled:

- 20 sites total (10 active intervention, 10 usual programming control)
  - *Approximately 40 after-school program staff (2 staff/site) (20 in the active intervention; 20 in the usual programming control)*
  - *Approximately 600 youth aged 11-14 years (300 in the active intervention; 300 in the usual programming control)*
  - *Approximately 10-20caregivers (1 parent/caregiver/active intervention site)*
- Source of Participants: All participants are being recruited from the beyond 3:30 after school program, which is a community organization that provides after-school programming to families in underserved communities across Toronto.
- Types of Recruitment: All 20 sites that operate beyond 3:30 will be included in the trial, therefore, no initial recruitment strategies are required.

How potential participants will be identified and approached: beyond 3:30 program administration will send an information flyer to parents/caregivers informing them about the EMPOWER SEL curriculum being implemented throughout programming and about the study that will take place over the 2024/2025 school year. Flyers will be sent to the Program Manager at beyond 3:30 who will then email the flyer to all parents who have children aged 11-14 years registered in the program.

- Facilitators who are a part of the intervention arm will inform youth about the addition of the SEL content in beyond 3:30 programming at the start of the school year.
- Staff Honorariums (staff are receiving more honorariums than caregivers and youth because we are asking them to complete more measures; all honorariums are based on a time metric of $30/hour)
  - Baseline, pre-intervention data collection: $30 e-gift card of choice
  - During Intervention: Monthly draw for 2 x $50 e-gift card of choice
  - Post-Intervention data collection: $30 e-gift card of choice
- Caregiver Honorariums
  - Post-Intervention Interview: $15 gift card of choice
- Youth Honorariums
  - Baseline, pre-intervention data collection: Pizza Party for each site
  - Post-Intervention: Pizza Party for each site

## 4.3 Equity, Diversity and Inclusion Considerations

The EMPOWER project, a community-academic partnership between CAMH and two after-school programs - beyond 3:30 and Visions of Science - is built on the foundations of equity. The EMPOWER project is a community-based participatory research endeavor to bolster the mental well-being of youth at participating after-school programs. Both beyond 3:30 and Visions of Science operate their programs in social and economically vulnerable neighbourhoods, many of which are within Toronto’s Neighbourhood Improvement Areas, which have disproportionate representation of racialized and marginalized individuals (City of Toronto, 2024). We have worked collaboratively to create four guiding project principles that inform all aspects of our work. These include utilizing (1) strengths-based approaches, (2) systemic understandings of socio-political structures that enable and hinder mental well-being, (3) youth-centered approaches, and (4) leveraging the strengths within each partner organization (Balter et al., 2024). All principles align with anti-Black and anti-oppression paradigms (Baah et al., 2019). These foundations of the project are represented in every research and practice endeavor.

For this pilot study, we’ve taken the following steps and considerations that are in alignment with EDI principles:

- Study Design Choice: The study design was chosen collaboratively between members of the CAMH team (Brendan Andrade, Alice-Simone Balter, Clement Ma, Madison Moloney) and beyond 3:30 administrative staff (Sandra Pierre and Sheldon Parkes). Providing all 20 sites access to the social-emotional curriculum was an explicit objective of beyond 3:30 administration and a primary factor within discussions of study design.
- Measurement Choice: A focus on strengths-based measures was emphasized as part of our inclusion criteria upon a review of measures for this pilot study. Collaborative reviews and discussions of potential measures were a part of a collaborative process between CAMH and beyond 3:30 and Visions of Science to decide on the strengths-based measures that are incorporated into this study.
- Youth Data Collection Procedure: All measures chosen for youth participants have been designed and used with youth and have appropriate age-and-grade readability. With youth-centeredness and inclusion in mind, we have built youth data collection procedures to ensure youth’s comprehension. Two CAMH staff will go to each respective site at the pre- and post-intervention time points to read aloud the survey questions to the whole class of youth. Research indicates that reading questionnaires aloud to youth results in better data quality compared to self-administration and this means of administration can be particularly beneficial for at-risk youth who may struggle with reading (Gresch et al., 2016).

## 4.4 Eligibility Criteria

### 4.4.1 Inclusion Criteria

Youth participants must meet all the inclusion criteria to be eligible to participate in completing measures for this this clinical trial:

1. Must be a youth aged 11-14 years attending the beyond 3:30 program
2. Must sign and date the informed consent form

Staff participants must meet all the inclusion criteria to be eligible for this clinical trial:

- - - 1. Must be a facilitator of the beyond 3:30 program
      2. Must sign and date the informed consent form

Caregiver/parent participants must meet all the inclusion criteria to be eligible for this clinical trial:

Must be a caregiver/parent of a youth aged 11-14 years attending the beyond 3:30 program. Caregivers/parents whose children decide not to participate in data collection are still able to participate in an interview post-SEL curriculum implementation.

Must sign and date the informed consent form

### 4.4.2 Exclusion Criteria

An individual who meets any of the following criteria will be excluded from participation in completing measures for this clinical trial:

- Youth within the beyond 3:30 program who are not 11-14 years.
- Caregivers/parents who have children younger than 11-14 years within the beyond 3:30 program.

## 4.5 Lifestyle Considerations

*Not applicable*

## 4.6 Screen Failures

*Not applicable*

## 4.7 Participant Withdrawal Criteria

### 4.7.1 When and How to Withdraw Participants

Participants are free to withdraw from completing measures for the clinical trial at any time.

The reason for participant discontinuation or withdrawal from the study will be recorded within the participant’s research record. Participants who withdraw from the trial prematurely will not be replaced. Withdrawal from the study means that staff or youth withdraw their participation in data collection, not implementation or participation in the SEL programming. If staff withdraw from their participation in any of the pre-, post-, weekly fidelity, or monthly feasibility data collection time points they are being asked to participate in, this withdrawal will not impede the delivery of the SEL programming as this will be implemented regardless. Likewise, if youth withdraw from their participation in any of the pre- and post- SEL curriculum implementation data, their participation in the SEL programming won’t be affected as this will be implemented regardless.

### 4.7.2 Follow-up for Withdrawn Participants

Any staff, youth, or caregiver participant can withdraw their consent to participate in data collection and can also request the withdrawal of their data.

For staff and youth, withdrawal of consent to participate in data collection and the withdrawal of data from the clinical trial only means that they will not participate in data collection measures; participation in the implementation and participation of the SEL programming will continue. Once withdrawn from the clinical trial, no further research procedures or evaluations will be performed, or additional research-specific data collected on the participant. Reasons for withdrawal will be recorded within the participant’s research record.

###### 4.7.3 Early Termination Visit

*Not applicable*

###### 4.7.4 Participants who are Lost to Follow-up

Lost to follow-up in this study relates only to staff participants who do not complete the data collection measures. Lost to follow-up cannot apply to youth (who are completing measures in-person) and caregivers/parents (who are participating in one post-SEL curriculum implementation interview). A staff participant will be considered lost to follow-up if they fail to complete survey measures after three reminders and cannot be reached by the research team.

- Before a staff participant is deemed lost to follow-up, the research team will make every effort to regain contact with the participant (where possible, three e-mails). These contact attempts should be documented in the participant’s research record.
- Should the staff participant continue to be unreachable, they will be considered to have withdrawn from the clinical trial with a primary reason of lost to follow-up.

# 5 STUDY INTERVENTION

## 5.1 Description

Project EMPOWER’s SEL curriculum has been co-designed by our partnership collective (CAMH, beyond 3:30, and Visions of Science) for use with youth aged 11-14 years to help build their social emotional competencies and improve their mental wellness. The curriculum is based on the five core SEL skills within the Collaborative for Academic, Social and Emotional Learning’s (CASEL; 2024) framework. These five competencies include: self-awareness, self-management, social awareness, relationship skills and decision-making and directly inform the domains we use in our curriculum: self-awareness, self-management, social awareness & relationship skills (have been collapsed together to reduce redundancy of lesson plan topics between these domains), and decision making. Each domain contains 4 lesson plan topics, and it takes 4 weeks (1 topic/week) to implement each domain. The complete curriculum takes 16 weeks to implement. The following chart details the lesson plan topics under each domain.

| **DOMAINS** | **WEEK 1** | **WEEK 2** | **WEEK 3** | **WEEK 4** |
| --- | --- | --- | --- | --- |
| **Self-Awareness** | The relationship between thoughts and feelings | Self-talk | Strengths and areas for development | Self-kindness |
| **Self-Management** | Identifying unhelpful thoughts | Evaluating unhelpful thoughts | Reframing unhelpful thoughts and evaluating change | Emotion regulation practice |
| **Social Awareness & Relationship Skills** | Perspective-taking | Empathy | Receiving feedback | Assertiveness |
| **Decision Making** | IDEA: Identifying the problem | IDEA: Describe choices for solving a problem | IDEA: Explore pros and cons of choices | IDEA: Act on a choice |

Each lesson plan topic has been designed to complement existing after-school program activities with easy-to-embed 5–7-minute lesson plans that center SEL. Facilitators retain the flexibility to decide which existing program activity to integrate each SEL topic into and how to teach each SEL topic. The curriculum structure requires each weekly topic to be embedded into two separate and unrelated program activities each week, and to also informally bring it into other program activities when it makes sense to do so, thus increasing youth’s exposure to the weekly SEL topic.

Incorporating the SEL topics into existing program activities maintains the program culture and approach and is an attempt to integrate SEL skills that are often needed to complete a task or activity successfully without the entire lesson focusing on the SEL skill exclusively. The focus on SEL skills are within the context of program activities (e.g. self-talk during a basketball game). With repeated exposure to the lesson plan topics each week, we anticipate that, over time, youth will begin to generalize and extend the SEL skills learned to other areas of their lives.

## 5.2 Treatment Regimen

The intervention will take 16 weeks and be delivered at beyond 3:30 sites. We have designed a 16-week curriculum which covers one topic per week based on feedback received from a preliminary trial of one domain of the SEL curriculum (see section 2.3 Clinical Data to Date), where facilitators informed that teaching two topics per week was too rushed and could be overwhelming for youth. As such, we extracted the most important topics within each domain and ensured that the topics were not redundant between domains.

## 5.3 Method for Assigning Participants to Treatment Groups

Study sites will be randomized 1:1 to receive either active intervention or usual programming. Covariate constrained randomization (Moulton, 2004) will be used to balance treatment allocation across two site-level characteristics: (1) anticipated class size (ordinal measure), and (2) whether facilitators who were a part of the iterative trial in May 2024 (binary measure). Given the relatively small number of sites and 3-site level characteristics, standard stratified randomization is not appropriate to balance sites as it may lead to incomplete strata (Ivers et al., 2012).

Covariate-constrained randomization. Briefly, since the site-level covariates will be known prior to the start of the study, we will calculate the covariate imbalance in the two site-level characteristics for all possible treatment allocations of the 20 sites. We then select a subset of treatment allocations that minimizes the covariate imbalance. In order to maintain randomness, the actual treatment allocation will be selected from this selected subset. We will implement the covariate constrained randomization in R using the software provided in Carter and Hood (2008).

## 5.4 Administration of Study Intervention

Prior to implementing the SEL curriculum, facilitators in the active intervention arm will require training. Training will take place after randomization occurs. Facilitators in the intervention arm will receive training at an agreed upon date in fall 2024. Initial training will be delivered in-person. Facilitators will not be compensated for their time in the training, but will be offered an honorarium to complete post-training questionnaires. Facilitators in the intervention arm only will have the opportunity for continued training in our weekly coaching sessions.

Once facilitators receive training, they will be able to implement the SEL lesson plan topics into their existing program activities twice each week at the timelines allotted for the intervention (fall 2024).We are asking facilitators in the intervention arm to complete weekly fidelity checklists to monitor adherence and adaptations to the curriculum and will host weekly coaching sessions with facilitators to support their administration of the SEL curriculum intervention.

## 5.5 Participant Compliance Monitoring

Facilitators in the intervention arm will be asked to complete weekly fidelity checklists which include a question assessing whether youth are engaged in the SEL lessons. We have structured weekly coaching sessions with facilitators (in the intervention arm) to discuss any challenges they may be having with unengaged youth.

## 5.6 Concomitant Therapy

Not applicable

## 5.7 Packaging

Not applicable

## 5.8 Blinding of Study Intervention

Not applicable

## 5.9 Receiving, Storage, Dispensing and Return

### 5.9.1 Receipt of Study Intervention Supplies

Not applicable

### 5.9.2 Dispensing of Study Intervention

Not applicable

### 5.9.3 Return or Destruction of Study Intervention

Not applicable

# 6 RESEARCH PROCEDURES

## 6.1 Research Visits

- Baseline Visit: Collection of pre-SEL curriculum implementation data
  - Youth and staff from both active intervention and usual programming control arms will be asked to participate in this data collection time point.
  - Youth questionnaires:
    - Demographic questions (researcher-created)
    - SSIS-SEL Brief Scale (Elliot et al., 2020)
    - KIDSCREEN (Child Public Health, 2024)
    - CD-RISC 10 (2007)

Procedure of youth data collection: Two CAMH research staff who meet the requirements of delivering consent procedures (e.g., cannot be an undergraduate student, must have proper training) will go to each of the 20 sites and (1) verbally go through the consent procedure with each class, (2) after the consent form is read to the class, CAMH research staff will check-in with each youth to ask them if they have any questions they can clarify about consent, (3) ask each youth to sign the consent form should they wish to proceed with participating in the completing the study measures (youth will sign these on tablets provided by CAMH, (4) data collection will begin, CAMH personnel will read aloud each question to the class while youth will record their answers on REDCap which will be set up on the tablet, (5) collect the tablets when everyone is finished, (6) provide a pizza party honorarium for their participation (every youth, regardless of whether they participate in the study or not, will be able to participate in the pizza party).

- - Staff questionnaires:
    - Demographic questions (researcher-created)
    - SSIS-SEL Brief Scale (Elliot et al., 2020)

KIDSCREEN 10 (Child Public Health, 2024Procedure of staff consent: Two CAMH research staff who meet the requirements of delivering consent procedures (e.g., cannot be an undergraduate student, must have proper training) will go to each of the 20 sites and (1) verbally go through the consent procedure with each facilitator, (2) ask each facilitator to sign the consent form via REDCap if they agree to participate, and (3) email each facilitator a signed copy of their completed consent form, (3) once consent is received from each staff member, we will email them a secure link to complete their survey online via REDCap, and (4) they will receive a $30 e-gift card of their choice for participating in the survey.

- 1. The pre and post measures will not contain any identifying information, such as names. Each youth participant will be assigned a study ID at the beginning of the study, which will be associated with their REDCap surveys. Youth and facilitators will be sent a unique link for each youth, where youth and facilitators will complete surveys. Study IDs will be linked to youth participant demographic information in a separate decoder document to which only research staff will have access. The file will be password-protected and saved on the CAMH network. Facilitators will be provided with information that links each youth ID to a youth’s name in a Word document via a secure data transfer. No other information about the youth apart from their ID and name will be part of this data. Facilitators will be able to save this document for use while they are completing youth questionnaires, but will be told to delete the file when they are finished completing the youth surveys. Facilitators will only complete surveys for youth aged 11-14 years registered in their site who have consented to participating in the study.
- Weekly Fidelity Checklists, Data Collection
  - Staff in the active intervention arm will be asked to complete a weekly fidelity checklist for the duration of the SEL curriculum implementation.

Procedure of staff data collection: Automatic invites will be set up on REDCap and sent to facilitators to complete the weekly fidelity checklist online via REDCap. The honorarium for completing the weekly fidelity checklist is a monthly draw for, for 16 weeks, for 2 X $50 e-gift card of choice.

- Weekly Coaching Sessions (this is offered to staff to support their implementation of the EMPOWER SEL curriculum)
  - There will be 2 weekly coaching sessions (with 10 facilitators in each group) held that staff in the active intervention arm will be asked to attend. These coaching sessions will be scheduled online via Webex. Notes containing no personal identifying information will be taken to document these meetings. Should facilitators be unable to attend, they will be sent the meeting notes to review via email. The weekly coaching sessions are held to maintain fidelity to the intervention and provide facilitators with ongoing support and guidance. Meeting notes are taken so that facilitators and program staff can refer back to information that was discussed. As such, if a facilitator misses a meeting they will be sent meeting notes in order to provide them with information about discussion and support fidelity. There is no honorarium for the weekly coaching sessions. Staff will be sent a Webex meeting invite weekly for a 30-minute session.
- Monthly Feasibility Measures, Data Collection
  - Staff in the active intervention arm will be asked to complete three feasibility measures every 4 weeks, after each SEL domain has been taught.
    - Feasibility of Intervention Measure (Weiner et al., 2017)
    - Acceptability of Intervention Measure (Weiner et al., 2017)
    - Intervention Appropriateness Measure (Weiner et al., 2017)

Procedure of staff data collection: We will email staff a secure link to complete the survey online via REDCap. The honorarium for completing the feasibility measures each month is a monthly draw for, for 16 weeks, for 2 X $50 e-gift card of choice.

- Post-SEL curriculum implementation: Data Collection
  - Youth and staff from both active intervention and usual programming control arms will be asked to participate in this data collection time point.
  - Youth questionnaires:
    - SSIS-SEL Brief Scale (Elliot et al., 2020)
    - KIDSCREEN (Child Public Health, 2024)
    - CD-RISC 10 (2007)

Procedure of youth data collection: Two CAMH research staff who meet the requirements of delivering consent procedures (e.g., cannot be an undergraduate student, must have proper training) will go to each of the 20 sites and (1) distribute tablets to each youth, (2) read aloud each question to the class while youth will record their answers on REDCap which will be set up on the tablet, (3) collect the tablets when everyone is finished, (4) provide a pizza party honorarium for their participation (every youth, regardless of whether they participate in the study or not, will be able to participate in the pizza party).

- - Staff questionnaires:
    - SSIS-SEL Brief Scale (Elliot et al., 2020)
    - KIDSCREEN 10 (Child Public Health, 2024)

Qualitative Questions (researcher created) Procedure of staff data collection: We will email staff a secure link to complete the survey online via REDCap. They will receive a $30 e-gift card of their choice for participating in the survey. The pre and post measures will not contain any identifying information, such as names. Each youth participant will be assigned a study ID at the beginning of the study, which will be associated with their REDCap surveys. Youth and facilitators will be sent a unique link for each youth, where youth and facilitators will complete surveys. Study IDs will be linked to youth participant demographic information in a separate decoder document to which only research staff will have access. The file will be password-protected and saved on the CAMH network. Facilitators will be provided with information that links each youth ID to a youth’s name via a secure data transfer. No other information about the youth apart from their ID and name will be part of this data. Facilitators will only complete surveys for youth aged 11-14 years registered in their site who have consented to participating in the study.

Procedure of parent data collection: Beyond 3:30 administration will identify at least 1 parent from each of the intervention arm sites they feel would be interested in participating in a 30-minute interview, thus we are adopting a convenience sampling approach. More than 1 parent representing more than one youth can participate. We are aiming to interview at least 10 parents. Beyond 3:30 staff will reach out to all parents in an email with an invitation to participate, in order to identify at least 1 parent at each of the 10 sites.

- - Beyond 3:30 administration will email parents an invitation to participate in a 30-minute study with a copy of the informed consent form attached. If the caregiver is interested in participating, a member of the study team will call the caregiver to go over the consent form. If the caregiver agrees to participate in the study, a virtual interview via Webex will be scheduled. During this interview, a member of the study team will ask the caregiver about their child’s social emotional skills. . Caregivers will receive a $15 gift card of their choice for participating in the interview.

## 6.2 Schedule of Events

| **Procedures** | Two weeks pre- SEL curriculum implementation | Week  1 | Week  2 | Week  3 | Week  4 | Week  5 | Week  6 | Week  7 | Week  8 | Week  9 | Week  10 | Week  11 | Week  12 | Week  13 | Week  14 | Week  15 | Week  16 | Two weeks post-SEL curriculum implementation |
| --- | --- | --- | --- | --- | --- | --- | --- | --- | --- | --- | --- | --- | --- | --- | --- | --- | --- | --- |
| **Pre-SEL Implementation Data Collection (participants in active and usual programming arms)** |  |  |  |  |  |  |  |  |  |  |  |  |  |  |  |  |  |  |
| Youth Consent | X |  |  |  |  |  |  |  |  |  |  |  |  |  |  |  |  |  |
| Facilitator Consent | X |  |  |  |  |  |  |  |  |  |  |  |  |  |  |  |  |  |
| Administration of Youth Survey | X |  |  |  |  |  |  |  |  |  |  |  |  |  |  |  |  |  |
| Administration of Staff Survey | X |  |  |  |  |  |  |  |  |  |  |  |  |  |  |  |  |  |
| **Administration of the SEL Curriculum (active intervention arm)** |  | X | X | X | X | X | X | X | X | X | X | X | X | X | X | X | X |  |
| **Fidelity Checklists (facilitators in active intervention arm)** |  | X | X | X | X | X | X | X | X | X | X | X | X | X | X | X | X |  |
| **Coaching Sessions (facilitators in active intervention arm)** |  | X | X | X | X | X | X | X | X | X | X | X | X | X | X | X | X |  |
| **Feasibility Measures for each SEL domain (facilitators in active intervention arm)** |  |  |  |  | X |  |  |  | X |  |  |  | X |  |  |  | X |  |
| **Post -SEL Implementation Data Collection (participants in active and usual programming arms)** |  |  |  |  |  |  |  |  |  |  |  |  |  |  |  |  |  | X |
| **Administration of Youth Survey** |  |  |  |  |  |  |  |  |  |  |  |  |  |  |  |  |  | X |
| Administration of Staff Survey |  |  |  |  |  |  |  |  |  |  |  |  |  |  |  |  |  | X |
| Parent/Caregiver Consent |  |  |  |  |  |  |  |  |  |  |  |  |  |  |  |  |  | X |
| Parent/Caregiver Interview |  |  |  |  |  |  |  |  |  |  |  |  |  |  |  |  |  | X |
|  |  |  |  |  |  |  |  |  |  |  |  |  |  |  |  |  |  |  |
|  |  |  |  |  |  |  |  |  |  |  |  |  |  |  |  |  |  |  |

# 7.0 STATISTICAL PLAN

## 7.1 Sample Size Determination

**Sample size considerations**

Sample size and power calculations were performed using Power Analysis and Sample Size Software (PASS; 2024).

Co-primary endpoint 1: Feasibility

Feasibility will be assessed by facilitators in the active intervention arm. We expect N=20 facilitators in total (10 active sites x 2 facilitators per site). A sample size of 20 facilitators achieves an exact binomial 95% confidence interval width = 42.3% for co-primary endpoint 1, assuming the intervention is feasible for 14 out of 20 (70%) facilitators.

Co-primary endpoint 2: Social emotional learning

A sample size of 20 clusters (10 active, 10 usual programming) with an average of 20 youth per cluster achieves 89% power (α=5%) to detect a small to medium effect size of 0.35 between arms in the primary endpoint. We assume the coeffient of variation for the cluster sizes is assumed to be 0.25, and the intracluster correlation (ICC) is 0.01.

## 7.2 Statistical Methods

1. Co-primary Objectives: Feasibility

   Acceptability, Feasibility, and Appropriateness of Intervention Measures: Descriptive statistics will be used to summarize acceptability, feasibility, and appropriateness of intervention measures. Frequencies and proportions will summarize categorical measures; means, medians, standard deviations and ranges will summarize continuous measures.

The frequency and proportion of facilitators and youth who “completely agree” or “agree” that the SEL curriculum is feasible, acceptable, and appropriate will be calculated, along with exact binomial 95% confidence intervals. We’ve set our progression criteria threshold to 70% for feasibility measures. This means that 70% of facilitators and youth “completely agree” or “agree” that the SEL curriculum is feasible, acceptable, and appropriate. We use a traffic light approach (Mellor et al., 2021) where meeting the set threshold equates to a green light and that the curriculum can proceed into its next phases of testing. Changes to the SEL curriculum are required should the threshold not be met (50%-60% = yellow light, or under 50% = red light). In these cases, a consensus meeting with the EMPOWER collaborative (partners [beyond 3:30 and VOS] and staff, youth, and caregiver advisors) will follow to determine necessary revisions and determine the best way to move forward.

Fidelity checklists: Each of the constructs within the fidelity checklist are measured in one-item and are descriptive in nature. They will be analyzed as such.

1. Co-primary Objectives: Social-Emotional Learning
2. SSIS-SEL Brief Scale

The unit of analysis for the primary comparison is individual youth. Linear mixed models will be used to compare pre-post intervention SSIS-SEL scores between groups assuming the fixed effects of treatment group (EMPOWER vs. Usual Programming), time (0 and 18 weeks post-initiation of intervention, and treatment-by-time interaction. To account for repeated measures, random effects of site and youth will be included in the model.

1. Secondary Objectives (secondary)
2. KIDSCREEN and CD-RISC 10 Measures

A similar statistical approach as for co-primary endpoint 2 (SEL) will be used to compare secondary endpoints between arms.

# 8.0 SAFETY AND ADVERSE EVENTS

## 8.1 Definitions

Not applicable

## 8.2 Recording of Adverse Events

We do not anticipate any adverse events for youth, staff, or parents who participate in completing study measures. However, any adverse events will be immediately reported to the REB.

## 8.3 Reporting of Serious Adverse Events

### 8.3.1 Investigator Reporting: Notifying the Sponsor

Not applicable

### 8.3.2 Investigator Reporting: Notifying the REB

Not applicable

### 8.3.3 Sponsor Reporting of Adverse Events: Notifying Health Canada

Not applicable

### 8.3.4 Sponsor Reporting of Adverse Events: Notifying Sites

We do not anticipate any adverse events for youth, staff, or parents who participate in completing study measures. Any adverse events will be addressed on a site-by-site basis.

## 8.4 Reporting of Device Deficiencies

Not applicable

## 8.5 Safety Management Plan

- Youth safety and minimizing experiences of distress during youth involvement in the SEL curriculum will be monitored by beyond 3:30 staff who are already trained to identify youth distress within the context of the beyond 3:30 program. Facilitators will inform their beyond 3:30 administrators as well as the CAMH research team should any youth experience distress during the SEL curriculum implementation. Resources for mental health support will be shared with the youth participant and their parent/caregiver.

## 8.6 Unblinding Procedures

Not applicable

## 8.7 Data and Safety Monitoring Board

Not applicable

# 9.0 CLINICAL TRIAL DISCONTINUATION AND CLOSURE

## 9.1 Clinical Trial Discontinuation

This clinical trial may be temporarily suspended or prematurely terminated if there is sufficient reasonable cause (i.e. closure based on PI decision, sponsor/funder decision, REB or other oversight bodies’ decision; review of serious, unexpected and related AEs; noncompliance; futility). Notification, which includes the reason for study suspension or termination, will be provided by the suspending or terminating party to research participants, the PI, funding agency, CAMH, and regulatory authorities. If the clinical trial is prematurely terminated or suspended, the PI will promptly inform research participants, the REB, and the sponsor, and will provide the reason(s) for the termination or suspension. All communication with participants for this purpose will go through REB review and approval. Research participants will then be contacted, as applicable, and be informed of changes to the study visit schedule.

# 10.0 DATA HANDLING AND RECORD KEEPING

## 10.1 Source Documents & Case Report Forms

Data for this clinical trial will be managed using REDCap electronic case report forms. This system is maintained on central CAMH servers, with data backed up daily, and is supported by the Research Informatics department.

## 10.2 Study Equipment Calibration Records

Not applicable

## 10.3 Protocol Deviations

No deviations from or changes to the protocol will be implemented without prior agreement from the sponsor as required, and approval from the REB, unless it is to eliminate an immediate hazard to a participant.

## 10.4 Record Retention

The PI will maintain all records pertaining to this clinical trial for 10 years. The 10-year period complies with the Public Hospitals Act retention requirements of patient PHI for non-regulated research. This is also consistent with retention requirements for major journals.

## 10.5 Clinical Trial Registration

This clinical trial will be registered in a publicly accessible database ([www.clinicaltrials.gov](http://www.clinicaltrials.gov)) once REB approval has been attained.

# 11.0 STUDY MONITORING, AUDITING, AND INSPECTING

## 11.1 Study Monitoring Plan

Site monitoring is conducted to ensure that the rights and well-being of research participants are protected, the reported trial data are accurate, complete, and verifiable, and the conduct of the clinical trial is in compliance with the currently approved protocol/amendment(s), ICH GCP, and applicable regulatory requirement(s).

- Facilitator and site monitoring will occur throughout the trial to ensure the intervention is being implemented correctly and that all research measures are completed as intended. Facilitator monitoring will occur via virtual coaching sessions weekly for the entire trial. The monitoring will occur via Webex meetings.
- Independent audits may be conducted by research staff to ensure monitoring practices are performed consistently across all participating sites.

## 11.2 Auditing and Inspecting

The PI and site will permit study-related audits, and inspections by the REB, CAMH, sponsor, and applicable granting agencies or regulatory bodies, including access to all study-related documents (e.g. source documents, regulatory documents, data collection instruments, study data, etc.). The PI will ensure the capability for audits/inspections of applicable study-related facilities (e.g. research pharmacy, clinical laboratory, imaging facility, etc.).

# 12.0 ETHICAL CONSIDERATIONS

## 12.1 Research Ethics Board (REB) Approval

Research Ethics Board (REB) approval will be obtained prior to beginning any research-specific procedures. Following initial ethics approval, ongoing ethical approval will be maintained, and the clinical trial will undergo REB review at least annually, in accordance with regulatory and REB requirements. The clinical trial will be conducted in accordance with the REB-approved study documents and the determinations (including any limitations) of the REB, and in compliance with REB requirements.

Whenever new information becomes available relevant to participant consent, a consent form and/or consent for addendum will be presented to the REB for review and approval before its use. Any revised written information will receive REB approval prior to use.

## 12.2 Informed Consent Process & Documentation

All participants will be asked to consent using electronic consent. Electronic consent will be obtained from participants using the REDCap e-Consent framework developed by CAMH.

Participants invited to participate in the research study will be asked to provide Informed Consent prior to completing any research measures. During the review of the Consent Form, trained senior lab members will explain the purpose of the research study, the treatment intervention, the risks and benefits to participating, the expected duration of the subject’s participation, the subject’s responsibilities, the compensation for participating, confidentiality and privacy, and that all participation is entirely voluntary. The individual obtaining consent must also indicate that if participants do choose to withdraw, it will in no way affect their relationship with beyond 3:30.

If participants agree to participate in the study, they are asked to sign and initial where appropriate only after the Consent Form has been reviewed in great detail. If participants do not feel as though they thoroughly understand every component of the research project, the person obtaining consent must expand on any unclear sections. All questions should be answered during the consent process, and any misunderstandings clarified.

Parents will be asked to provide consent for the following:

- Read the consent form and have been informed of the purpose of the research
- Consent to participate in the research study
- Consent to contact for future research
- Consent to de-identified data being used for other research

Facilitators will be asked to provide consent for the following:

- Read the consent form and have been informed of the purpose of the research
- Consent to participate in the research study
- Consent to contact for future research
- Consent to de-identified data being used for other research

Youth aged 11-14 years will also be asked to provide consent. We lean on the evidence from Hein et al. (2014) who assessed the optimal cut-off ages for young people to consent to medical decision making, which was 10.4 years. Given that we are seeking youth consent to participate in completing data measures, we are confident that youth aged 11-14 years are capable to provide consent. However, caregivers/parents will be provided with an information flyer about the study prior to the youth consent discussion.

Trained senior lab members will obtain this consent. Study information will be presented to youth as a group and then youth will be given ample and unlimited time to think about their participation in the study and review the consent form in a separate space that is different from the programming space. This separation from programming space will offer youth a quiet space with limited distractions to consider their participation in the study. Youth will be invited by a CAMH staff member and program staff member to an individual meeting outside of the group (e.g. private space) to discuss any questions or concerns they may have regarding the study. Youth will be supported by staff members who they are familiar with and can ask any questions they may have. During the individual meeting, it will be emphasized to youth that their participation is voluntary and they are able to withdraw at any time. Further, that not participating in the study will in no way impact their ability to participate in the larger beyond 3:30 program. This information will remain confidential. Youth can choose to take the consent form home and complete the consent process at a later date.

- Youth are asked to sign where appropriate only after the Consent Form has been reviewed in detail. If youth do not feel as though they thoroughly understand every component of the research project, the person obtaining consent must expand on any unclear sections. If youth do not consent they may leave the room and complete other program activities while those who do consent remain in the room to complete the data measures. A paper copy of the consent form will be given to youth for their records. . It is the responsibility of the senior lab member to explain the consent form using child-friendly vocabulary. If a youth does not wish to participate in completing data measures, that is their right. Youth who do not consent to completing data measures will still participate in the SEL curriculum, but not participate in the data collection session.

All youth will be provided with a paper copy of the consent form as we go through the document as a group. They will be able to keep this copy regardless of whether they consent to participate in the study or not. Staff will be given a paper copy of the consent form as we go through the document in-person. Parents will be sent a copy of their consent form via email and offered the option to be mailed a paper copy.

# 13.0 PRIVACY AND CONFIDENTIALITY

All clinical trial-related documents and data will be held in strict confidence and stored on CAMH servers and will follow CAMH policies and procedures to ensure participant privacy and confidentiality. Data is accessible by the CAMH research team only; IT at CAMH may have access to emails for the purposes of technical support. Data is encrypted and data is protected in the CAMH server. No information concerning the clinical trial or the data will be released to any unauthorized third party without prior written approval of the sponsor, and the consent of the participant (where applicable). Only aggregate data will be shared in these cases.

All research activities will be conducted in as private a setting as possible (youth will be in a group setting at their after-school program; facilitators will complete their surveys individually via REDCap; and, caregivers/parents will engage in a 1:1 interview via WebEx). The study monitor, other authorized representatives of the sponsor, representatives of the REB, regulatory agencies may inspect all documents and records required to be maintained by the PI. The participant’s contact information will be securely stored at CAMH for internal use during the clinical trial. At the end of the clinical trial, all records will continue to be kept in a secure location in accordance with applicable institutional and regulatory requirements.

At the beginning of the study, each youth participant will be assigned a study ID. As youth and facilitators are completing the pre- and post-intervention survey measures, a participant database that links the ID of each youth will be created. Youth and facilitators will be sent a unique link for each youth, where youth and facilitators will complete surveys. Facilitators will be provided with information that links each youth ID to a youth’s name via secure data transfer. No other information about the youth apart from their ID and name will be part of this data.

Because we wish to understand facilitator’s perceptions of fidelity and feasibility, and they will be completing measures on a weekly and monthly basis, they will also be assigned a participant ID which will be stored in the participant database. Only the CAMH research team will have access to this information. All files will be password-protected and saved on the CAMH network.

The research team will take notes during the weekly coaching sessions with facilitators for the purpose of maintaining fidelity to the intervention and providing facilitators with ongoing support and guidance and also used to refine the curriculum. These notes will not contain any identifying information and will be stored on the secure CAMH network.

# 14.0 CLINICAL TRIAL FINANCES

## Funding Source

This study is generously funded by the Les Lois Shaw Foundation.

## Conflict of Interest

Not applicable

# 15.0 PUBLICATION POLICY/DATA SHARING

Not applicable as this is not an NIH-Funded study.

## 15.2 Future Secondary Use of Data

De-identified data from this project may be used for future research by internal and/or external project collaborators. De-identified and anonymized data from this clinical trial will be deposited to CAMH’s BrainHealth Databank (BHDB) for potential future use by other investigators including those outside the clinical trial. Participant consent to share data collected from this clinical trial for future secondary research will be in accordance with BHDB policies and procedures.  The informed consent form details what the BHDB is and how data will be used once it is stored there. Participants are also informed of any risks or benefits to sharing their data with the BHDB and how their data will be protected.

# 16.0 REFERENCES

Arakelyan, M., Freyleue, S., Avula, D., McLaren, J. L., O’Malley, A. J., & Leyenaar, J. K. (2023). Pediatric mental health hospitalizations at acute care hospitals in the US, 2009-2019. *JAMA*, *329*(12), 1000-1011. <http://doi.org/10.1001/jama.2023.1992>

Baah, F. O., Teitelman, A. M., & Riegel, B. (2019). Marginalization: Conceptualizing patient vulnerabilities in the framework of social determinants of health—An integrative review. *Nursing inquiry*, *26*(1), e12268.

Balter, A., Racine, N., Al-Khooly, D., Simir, I., Bandoles, E., Utchay, C., Sylvestre, D., Sibilus, A., Suri, A., Pierre, S., Parkes, S., Quesnelle, S., Brodkin, S., & Andrade, B.F.  (2024). Strengthening youth emotional and behavioral well-being through community-academic partnership: The EMPOWER project. *Health Promotion Practice, 0*(0). <https://doi.org.10.1177/15248399241255372>

Carter, B.R., & Hood, K. (2008). Balance algorithm for cluster randomized trials. *BMC Medical Research Methodology, 8*, 65. 10.1186/1417-2288-8-65

Durlak, J. A., Mahoney, J. L., & Boyle, A. E. (2022). What we know, and what we need to find out about universal, school-based social and emotional learning programs for children and adolescents: A review of meta-analyses and directions for future research. *Psychological Bulletin*, *148*(11-12), 765.

Durlak, J.A., Weissberg, R.P., 7 Pachan, M. (2010). A Meta-Analysis of After-School Programs That Seek to Promote Personal and Social Skills in Children and Adolescents. *American Journal of Community Psychology, 45*, 294-309. <https://doi.org/10.1007/s10464-010-9300-6>

Durlak, J.A. (2016) Programme implementation in social and emotional learning: basic issues and research findings, *Cambridge Journal of Education, 46*(3), 333-345. https://doi.org/10.1080/0305764X.2016.1142504

Fante-Coleman, T., & Jackson-Best, F. (2020). Barriers and Facilitators to Accessing Mental Healthcare in Canada for Black Youth: A Scoping Review. *Adolescent Research Review, 5*, 115–136. <https://doi.org/10.1007/s40894-020-00133-2>

Frazier, S.L., Rusch, D., Coxe, S., Stout, T.J., Helseth, S.A., Dirks,M.A., Bustamante, E.E., Atkins,M.S., Glisson, C., Green, P.D., Bhaumik, D., & Bhaumik, R. (2021) After-School Programs and Children’s Mental Health: Organizational Social Context, Program Quality, and Children’s Social Behavior*, Journal of Clinical Child & Adolescent Psychology, 50*(2) 215-228. <https://doi.org/10.1080/15374416.2019.1683849>

Gresch, C., Strietholt, R., Kanders, M., & Solga, H. (2016). Reading-aloud versus self-administered student questionnaires: An experiment on data quality. In *Methodological Issues of Longitudinal Surveys: The Example of the National Educational Panel Study* (pp. 561-578). Wiesbaden: Springer Fachmedien Wiesbaden.

Kauhanen, L., Yunus, W.M.A.W. M, Lempinen, L., Peltonen, K., Gyllenberg, D., Mishina, K., Gilbert, S., Bastola, K., Brown, J. S. L., & Sourander, A. (2023). A systematic review of the mental health changes of children and young people before and during the COVID‑19 pandemic. *European Child & Adolescent Psychiatry (32), 995–1013* <https://doi.org/10.1007/s00787-022-02060-0>

Ivers, N.M., Halperin, I.J., Barnsley, J., et al., (2012). Allocation techniques for balance at baseline in cluster randomized trials: A methodological review. Trials, 13, 120. https://doi.org/10.1186/1745-6215-13-120

Madigan, S., Racine, N., Vaillancourt, T., Korczak, D. J., Hewitt, J. M., Pador, P., ... & Neville, R. D. (2023). Changes in depression and anxiety among children and adolescents from before to during the COVID-19 pandemic: A systematic review and meta-analysis. *JAMA Pediatrics*, *177*(6), 567-581. <https://www.doi.org/10.1001/jamapediatrics.2023.0846>

Mellor K, Eddy S, Peckham N, Bond, C.M., Campbell, M.J., Lancaster, G.A., Thabane, L., Eldridge, S.M., Dutton, S.J., & Hopewell, S. (2021). Progression from external pilot to definitive randomised controlled trial: a methodological review of progression criteria reporting. *BMJ Open,* 11:e048178. doi:10.1136/ bmjopen-2020-048178

Moulton, L.H. (2004). Covariate-based constrained randomization of group-randomized trials. Clinical Trials,

Moulton LH: Covariate-based2004, 1: 297-305.

Murthy, V.H. (2022). The mental health of minority and marginalized young people: An opportunity for action. *Public Health Reports, 137*(4), 613-616. <https://doi.org/10.1177/00333549221102390>

PASS (2024). Power Analysis and Sample Size Software (PASS; 2024). NCSS. LLC. Kaysville, Utah, USA. Ncss.com/software/pass

Polanczyk, G.V., Salum, G.A., Sugaya, L.S., Caye, A., & Rohde, L.A. (2015). Annual Research Review: A meta-analysis of the worldwide prevalence of mental disorders in children and adolescents. *Journal of Child Psychology and Psychiatry 56*(3), 345–365 <https://doi.org/10.1111/jcpp.12381>

Raphael D, Byrant T, Mikkonen J, Raphael A. *Social determinants of health: The Canadian facts*. 2020. <http://www.thecanadianfacts.org/>

Samji, H., Wu, J., Ladak, A., Vossen, C., Stewart, E., Dove, N., ... & Snell, G. (2022). Mental health impacts of the COVID‐19 pandemic on children and youth–a systematic review. *Child and adolescent mental health*, *27*(2), 173-189.

Schonert-Reichl, K. A., Kitil, M. J., & Hanson-Peterson, J. (2017). To reach the students, teach the teachers: A national scan of teacher preparation and social and emotional learning. A report prepared for the Collaborative for Academic, Social, and Emotional Learning (CASEL). Vancouver, B.C.University of British Columbia.

Taylor, R. D., Oberle, E., Durlak, J. A., & Weissberg, R. P. (2017). Promoting positive youth development through school‐based social and emotional learning interventions: A meta‐analysis of follow‐up effects. *Child development*, *88*(4), 1156-1171.
